# Short-term efficacy of peripheral nerve stimulation for essential tremor in a randomized double-blind controlled trial

**DOI:** 10.1038/s41598-025-13487-1

**Published:** 2025-08-06

**Authors:** Reza Samiee, Melika Jameie, Masoud Rahmati, Mehdi Azizmohammad Looha, Sheida Mobader, Abbas Tafakhori, Payam Sarraf, Hamed Amirifard, Sakineh Ranji Burachaloo, Mojdeh Ghabaee, Mobina Amanollahi, Zohreh Tajabadi, Mohammad Hossein Harirchian

**Affiliations:** 1 Iranian Center of Neurological Research, Neuroscience Institute, Tehran University of Medical Sciences, Tehran, Iran; 2 Amirkabir University of Technology, Tehran, Iran; 3 Basic and Molecular Epidemiology of Gastrointestinal Disorders Research Center, Research Institute for Gastroenterology and Liver Diseases, Shahid Beheshti University of Medical Sciences, Tehran, Iran; 4 School of Medicine, Tehran University of Medical Sciences, Tehran, Iran; 5 Digestive Disease Research Institute, Tehran University of Medical Sciences, Tehran, Iran

**Keywords:** Tremor, Action tremor, Essential tremor, Transcutaneous electric nerve stimulation, Quality of life, Neurology, Movement disorders

## Abstract

Peripheral nerve stimulation (PNS) demonstrates promise for some neurological conditions. However, its effectiveness for essential tremor (ET) requires further research. In this randomized, double-blind, sham-controlled trial, we investigated the safety and efficacy of a single, 40-minute PNS session in ET. Eighty-eight participants (age: 63.5 [52.5, 70.3], male: 57.95%) were assigned to active PNS stimulation (*n* = 45) or sham procedure (*n* = 43). Efficacy was assessed using accelerometer-measured tremor amplitude (m/s²), clinician-rated tremor severity using the Tremor Research Group Essential Tremor Rating Assessment Scale (TETRAS), patients’ own experience with daily activities using the Bain and Findley Activities of Daily Living scale (BF-ADL), and their perceived improvement based on the Clinical Global Impression-Improvement scale (CGI-I). Assessments were conducted at baseline, immediately post-stimulation, and at multiple intervals up to 90 min (extending to 24 h for subjective outcomes). Temporal changes in outcomes over time were evaluated using the Generalized Estimating Equation (GEE). The effects of group, time, and their interaction (group*time) on the outcomes were subsequently assessed using GEE. GEE analyses revealed significant group*time interaction for tremor amplitude (B = − 51.61, 95%CI [− 94.60, − 8.62], *p* = 0.019). However, no significant group*time interactions were observed for TETRAS, BF-ADL, and CGI-I. This study indicated the effect of PNS on reducing ET amplitude. ID: IRCT20161212031362N2.

**Supplementary Information:**

The online version contains supplementary material available at 10.1038/s41598-025-13487-1.

## Introduction

Action tremors considerably affect the quality of life^1,2^. Essential tremor is the most common neurological cause of action tremors, with a prevalence of up to 5% across the general population^1–5^. Beta-blockers and anti-seizure medications are often the first line of treatment, aiming to reduce hand tremor amplitude by around 50%^1,6–8^. However, these medications may lose effectiveness over time, and roughly a third of patients experience no significant benefit^7,8^. OnabotulinumtoxinA injections offer another option, providing relief for about three months but potentially causing muscle weakness^1^. For patients who do not respond well to medications, surgical interventions, including deep brain stimulation and focused ultrasound thalamotomy can be considered^6,9^. While offering remarkable tremor reduction, some patients might hesitate due to the nature of these procedures^6,9^.

Non-invasive, non-pharmacological approaches, including peripheral nerve stimulation (PNS), are attracting increasing interest in treating neurological conditions, including depression, epileptic disorders, and pain management^6,10–17^. The use of electrical stimulation for peripheral nerves to manage tremors has roots as far back as the 1980 s when Bathien et al. explored the effects of muscle twitching in essential tremor^18^. More recently, research has shifted towards investigating the stimulation of the median and radial nerves as a potential treatment for essential tremor^19–21^. This approach appears to work by modulating neural pathways involving the ventral intermediate nucleus of the thalamus (VIM), a region believed to be central to essential tremor pathology^19,22,23^.

While previous studies have explored the effects of PNS on essential tremor^19–21,24–26^, several key limitations persist. For instance, sham-controlled trials were limited to single-session designs, and within those sessions, they only assessed outcomes at two time points—baseline and immediately after stimulation—without evaluating sustained effects over time^19,20^. On the other hand, studies that investigated longer-term effects over multiple sessions lacked a control group^21,24,26^. Additionally, earlier research often excluded patients with mild tremors^20^, which might limit the generalizability of their findings to real-world populations. Building on these promising prior results^19–21,24–26^, we investigated the efficacy and safety of a single-session PNS among individuals with essential tremor at five distinct time points: before stimulation, immediately post-stimulation, and at 30, 60, and 90 min post-stimulation, with subjective outcomes additionally monitored for up to 24 h. Objective measures of efficacy included accelerometer-measured tremor amplitude (m/s²) and scores on a clinician-rated tremor scale. Subjective measures assessed patients’ own experience with daily activities, as well as their impression of improvement after stimulation. To our knowledge, this study is the first to investigate PNS for essential tremor in a Middle Eastern population. It also uniquely evaluates the durability of any potential improvements through multiple follow-up assessments after the stimulation period.

## Methods

### Trial design

Between September 2022 and March 2023, eighty-eight individuals with essential tremor participated in a parallel-group, randomized, double-blind, sham-controlled trial. With an allocation ratio of 1:1, they received a 40-minute active stimulation (intervention group) or sham procedure (non-intervention group), followed by a 90-minute follow-up period, extending to 24 h for subjective outcomes.

**Fig. 1:**
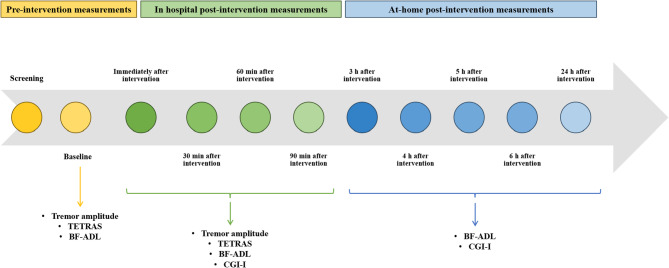
Participant flow diagram. *DBS* deep brain stimulator.

Figure 1 depicts the study design. During a screening visit, patient eligibility was determined. Those who qualified were scheduled to receive PNS or sham procedure one week later. Following a baseline assessment, participants received 40 min of either active stimulation or the sham procedure on their dominant hand. Immediately post-intervention, participants were evaluated again. Further in-hospital evaluations were conducted 30, 60, and 90 min after the intervention (in-hospital post-intervention stage). Participants were instructed to continue self-reporting subjective measurements at home (at-home post-intervention stage) at 3, 4, 5, and 6 h, as well as at 24 h after the intervention.

To minimize potential confounding variables, participants were instructed to abstain from caffeinated beverages (coffee, tea, cola drinks, cocoa) and avoid smoking and alcohol consumption throughout the study period, as these substances may influence tremor severity^27–29^. Concomitant tremor medications prescribed by their neurologists were allowed to continue. The engineering team determined the stimulation pattern for each patient under the supervision of a neurologist (please see the “Intervention” section for details). Two independent movement disorder specialists blinded to the intervention administered and scored the tremor assessments. All assessments were conducted by the same neurologists at baseline and each follow-up time point.

### Participants

Participants meeting the following criteria were included: (a) adults with essential tremor confirmed by a movement disorder specialist, (b) no satisfiable response to tremor medications, (c) if on tremor medication, the type and dose must have been stable for the past 3 months and during the study, (d) medications for other conditions must have been stable for the past 3 months and throughout the study, and (e) a score of one or higher on the Tremor Research Group Essential Tremor Rating Assessment Scale (TETRAS) at baseline evaluation or a score of two or higher on at least one task of the Bain and Findley Activities of Daily Living (BF-ADL) scale at baseline evaluation.

Exclusion criteria included (a) pregnancy, (b) severe liver or kidney disease, (c) implanted medical devices (pacemaker, defibrillator, deep brain stimulator), (d) other neurological conditions, including concomitant resting tremor, drug-induced tremor, epileptic disorders, moderate to severe peripheral neuropathy, or neurodegenerative diseases, (e) receiving OnabotulinumtoxinA toxin injection in hands within the past six months or a history of thalamotomy, (f) alcohol or caffeine consumption within 24 h of testing, (g) alcohol dependence or regular, high caffeine intake (> one daily serving for the past three months), (h) orthopedic plates in the forearm or compromised skin at the stimulation site, and (i) inability or unwillingness to participate or follow the study protocol.

### Study settings and ethics statement

This study was conducted at an academic hospital complex, affiliated with the Tehran University of Medical Sciences (TUMS), Tehran, Iran, and is reported according to the Consolidated Standards of Reporting Trials (CONSORT) guidelines^30^. The protocol was approved by the institutional review board (IR.TUMS.NI.REC.1401.039) and followed the ethical principles of the Declaration of Helsinki and Good Clinical Practice^31,32^. Following a thorough explanation of the trial, all participants provided written informed consent for participation and publication. All participants were informed that they had the right to withdraw from the trial at any time without affecting their therapy. The trial was registered in the Iranian Registry of Clinical Trials on 12/09/2022 (registration number: IRCT20161212031362N2), a Primary Registry in the WHO Registry Network.

### Intervention

Participants in the intervention group received 40 min of PNS delivered via a wearable wristband (Pishgaman Rah Salamat Pars). The stimulation was applied with the arm at rest. The sham group also wore the wristband for 40 min, with the arm at rest, without receiving electrical stimulation. Electrodes were positioned on the anterior surface of the wrist (over the median nerve) and the lateral aspect of the dorsal wrist (over the superficial radial nerve), with a single counter-electrode on the posterior surface. Given that the superficial radial nerve is purely sensory and the median nerve at the wrist contains both motor and sensory axons, stimulation amplitude was carefully increased to a level just below the motor threshold to avoid muscle contractions. This intensity is generally well tolerated by participants. The wristband was equipped with accelerometers to measure participants’ tremor frequency during a 20-second hand posture hold. The wristband collected raw acceleration data on three axes.

#### Tremor frequency assessment

To ensure robust frequency analysis, we utilized two complementary methods: auto-correlation and Fast Fourier Transform (FFT). Each method offers distinct advantages in analyzing the tremor data.

*Auto-correlation method*: Raw acceleration data from the wristband was processed using an auto-correlation algorithm to initially estimate tremor frequency. To refine the frequency estimate, we computed the Power Spectral Density (PSD) of the accelerometer data using Welch’s method with overlapping windows (2-second window with 50% overlap). The dominant frequency peak within the 4–12 Hz range, indicative of essential tremor^4^, was identified. Subsequently, the mean PSD within a ± 1.5 Hz window centered on the identified peak frequency was calculated across all accelerometer axes to obtain a final auto-correlation-based frequency estimate.

*Fast Fourier transform*: The accelerometer data was converted from the time domain to the frequency domain using FFT. This method decomposes the time-series signal into its constituent frequencies. FFT identified the amplitude (m/s²) and phase of various frequency components present in the signal. Dominant frequencies in the tremor signal were identified by peaks in the frequency spectrum.

#### Stimulation delivery

The tremor frequencies obtained from both the auto-correlation method and FFT analysis were compared to determine the final stimulation frequency. This subject-specific frequency was then used to program the stimulation waveform. The waveform consisted of a series of charge-balanced biphasic pulses delivered at specific parameters (frequency: 150 Hz, pulse width: 300 µs, inter-pulse period: 50 µs). The stimulation alternated between the median and radial nerves at the tremor frequency. A level slightly below the motor threshold was reached by gradually raising the stimulation amplitude.

### Study measures and outcomes

Baseline characteristics of participants were documented, including demographics, medical history, and tremor-related characteristics. Safety was evaluated by monitoring for skin irritation (redness, itching, swelling, or any discomfort at the contact site), paresthesia or pain, tremor worsening, or any other unsolicited adverse events after stimulation^20,21^. The efficacy outcome measures included: (a) tremor amplitude (as the main study objective, quantified by accelerometer (m/s²)), (b) clinician-rated TETRAS scores for tremor severity^33^, (c) patient-rated BF-ADL score for tremor severity^34^, and (d) subject self-reported assessment of improvement using the clinical global impressions scale of improvement (CGI-I)^35^. The efficacy outcome measures are described below:

*Tremor amplitude assessment using accelerometer data* (m/s^2^): Tremor amplitude was assessed using triaxial accelerometer sensors placed on the index finger of the dominant hand during a forward postural hold, recording acceleration along the X, Y, and Z axes. The sensors of the accelerometer were 16-bit devices with a sensitivity between − 2G and + 2G (G = 9.8 m/s^2^). To capture the dynamics of tremor, data were sampled at a sufficiently high frequency (e.g., 100 Hz), which is adequate for detecting tremor frequencies typically ranging from 4 to 12 Hz. Since raw accelerometer data often contain noise and motion artifacts unrelated to tremor, a band-pass filter was applied to isolate the frequency band associated with pathological tremor (4–12 Hz). After filtering, the Root Mean Square (RMS) of the signal was computed as a time-domain measure of signal magnitude.

$$\:RMS=\sqrt{\frac{1}{N}\sum\:_{i=1}^{N}{x}_{i}^{2}}$$

Where $\:{x}_{i}$ is the filtered acceleration value at sample $\:i$, and $\:N$ is the total number of samples.

To further characterize the temporal variation of tremor amplitude, the Hilbert Transform was applied to the filtered signal along the Z-axis to construct the analytic signal:

$$\:z\left(t\right)=x\left(t\right)+j\widehat{x}\:\left(t\right)$$

Where $\:x\left(t\right)$ is the filtered real signal, $\:\widehat{x}\:\left(t\right)$ is the Hilbert Transform of $\:x\left(t\right)$, and $\:z\left(t\right)$ is the complex analytical signal.

The instantaneous amplitude (i.e., the amplitude envelope) was then calculated as:

$$\:A\left(t\right)=\:\left|z\left(t\right)\right|=\:\sqrt{{x}^{2}\left(t\right)+\:{\widehat{x}}^{2}\left(t\right)}$$

This envelope reflects the time-varying magnitude of tremor oscillations and serves as a refined estimate of tremor amplitude.

*TETRAS*: TETRAS is an objective standardized clinical assessment tool used to evaluate tremor severity^33,36^. TETRAS hand tasks were assessed across various activities, including (1) forward outstretched posture, (2) lateral “wing beating” posture, (3) kinetic finger-nose-finger testing, (4) Archimedes spiral task, (5) handwriting, and (6) dot approximation^33,36^. Each task receives a score between 0 and 4 and higher scores indicate a higher severity of tremor. Please see Supplementary Table S1 for details.

*BF-ADL*: BF-ADL is a 25-item self-rated scale used to assess tremor’s impact on a participant’s ability to perform daily activities^34,37^. A subset of eight tasks that could be performed with one hand (unilaterally) were assessed in this study, including (1) using a spoon to drink soup, (2) cup holding, (3) pouring milk from a bottle, (4) dialing a telephone, (5) picking up change, (6) inserting an electric plug, (7) unlocking a door with a key, and (8) letter writing. Participants rated their performance on a 4-point scale, where: 1 = able to do without difficulty, 2 = able to do with some effort, 3 = able to do with a lot of effort, 4 = unable to do without assistance^34,37^.

*CGI-I*: CGI-I is a 7-point scale used to assess a patient’s self-reported improvement in their tremor severity compared to baseline^35^. The scale ranges from 1 to 7, where 1 = very much improved, 2 = much improved, 3 = minimally improved, 4 = no change, 5 = minimally worse, 6 = much worse, and 7 = very much worse^35^.

### Randomization

Participants were randomly assigned in a 1:1 ratio to either the intervention group receiving patterned stimulation or the non-intervention group receiving sham procedure. Block randomization with a block size of three was employed using Sealed Envelope | Randomisation (randomization) and online databases for clinical trials.

### Blinding

This study employed double-blinding to minimize bias. The calibration procedure was performed for both groups. Subsequently, all participants wore the device, while unaware of whether they received the actual stimulation or a sham procedure. Outcome assessments at baseline and follow-up points were performed for all participants. Participants were informed that stimulation might feel different for different people, or that some levels might be imperceptible, which helps manage expectations. The visual features of the device were similar between the groups to ensure isolating the specific effects of PNS from potential benefits associated with simply wearing the device, such as attention or a placebo effect. The independent neurologists who assessed the outcomes were blinded to the participant’s group allocation.

### Adherence monitoring

During stimulation and between evaluation periods, participants remained in a controlled environment under physician supervision. This ensured protocol compliance and prevented the consumption of substances like smoking, alcohol, or coffee. Before discharge, participants received detailed instructions on completing follow-up tasks at designated home endpoints. Additionally, they were encouraged to contact the investigator with any questions throughout the study. Finally, participants were contacted 24 h after stimulation for further data collection.

### Safety monitoring

Safety monitoring involved recording adverse events using a standardized checklist and open-ended questions, both during and up to 24 h after stimulation. Participants were encouraged to report any unexpected symptoms.

### Study size

The sample size was calculated based on three primary objectives: detecting a group × time interaction effect, evaluating within-group changes over time, and comparing CGI-I scores between groups at 24 h. For the first objective, a repeated measures design formula for continuous outcomes was used:

n = [2 × (Z_1_-_β_ + Z_1−α/2_)² × σ² × (1 − ρ)]/(Δ² × T × ρ), where σ² is the outcome variance, ρ is the intra-class correlation, Δ is the expected difference in slope, and T is the number of time points. Assuming σ² = 1, ρ = 0.5, Δ = 0.4, T = 5, 80% power, and α = 0.05, the required sample size was 20 per group (40 total). For within-group comparisons, the formula n = [(Z_₁−β_ + Z_₁−α/2_)² × σ_d_²]/Δ² was applied, with σ_d_ = 1.2 and Δ = 0.5, resulting in 46 participants. For the CGI-I comparison between groups at 24 h, the formula *n* = 2 × [(Z_₁−β_ + Z_₁−α/2_)² × σ²]/Δ² was used, assuming σ = 1.1 and Δ = 0.65, yielding 45 participants per group. All parameter estimates (e.g., variances and expected differences) were derived from preliminary pilot data (*N* = 10) collected during the initial phase of this study.

### Statistical analysis

Numerical variables were presented as either mean ± standard deviation (SD) or median (interquartile range (IQR)), and categorical variables were expressed as frequency (percentage). Statistical comparisons of numerical variables between treatment groups were conducted through Independent-Samples T-tests or the Mann-Whitney U test. The relationship between categorical variables and the intervention was assessed using Fisher’s exact test. Temporal changes in outcomes over time were evaluated using the Generalized Estimating Equation (GEE). Subsequently, the effects of group, time, and their interaction (group*time) on the outcomes were also assessed using GEE. Dunn’s test was employed for pairwise multiple comparisons of outcomes between intervention and non-intervention groups at each time point, with Bonferroni correction. Pairwise Wilcoxon comparisons with Bonferroni correction were employed for outcomes to assess the mean differences between time points across intervention and non-intervention groups. All analyses were conducted in R (version 3.4.2), and p-values less than 0.05 were considered statistically significant.

## Results

### Baseline demographic, clinical, and essential tremor-related characteristics of participants

**Fig. 2:**
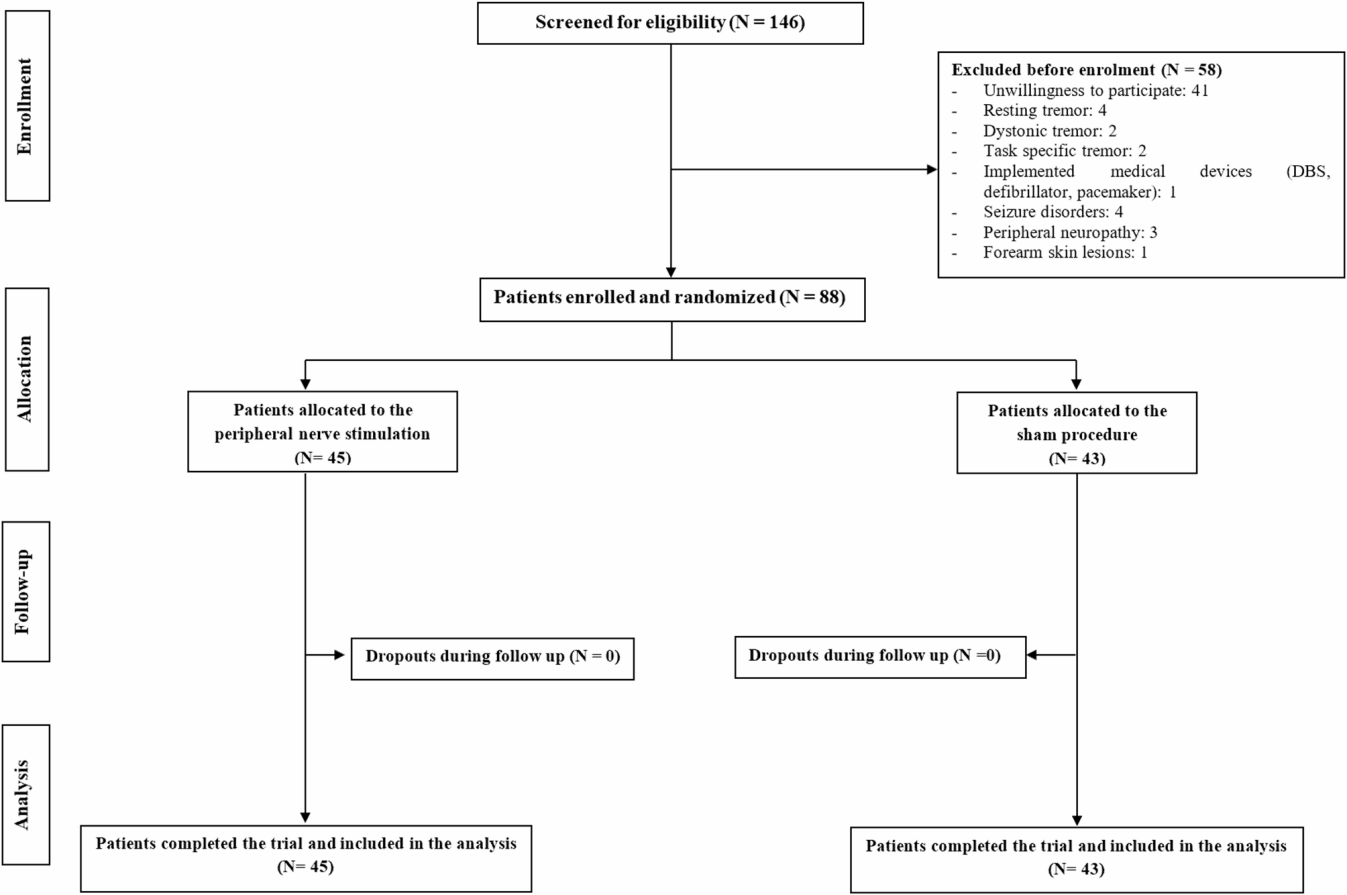
Study schedule and design. *BF-ADL* Bain and Findley Activities of Daily Living, *CGI-I* Clinical Global Impression-Improvement, *TETRAS* Tremor Research Group Essential Tremor Rating Assessment Scale, *min* minute(s), *h* hour(s).

Figure 2 depicts the participant flow throughout the study. A total of 146 individuals were screened for eligibility. Of these, 88 patients were enrolled and randomly assigned to either the intervention group (*n* = 45) or the non-intervention group (*n* = 43).

Table 1 presents participants’ baseline characteristics. The median age of participants was 63.5 [IQR: 52.5, 70.3] and 57.95% were male. The median disease duration was 5.0 years [3.0, 12.8]. Most participants (72.73%) were taking medications for their tremor, with a median treatment duration of 18.0 months [1.5, 36.0]. The two groups were comparable across their baseline demographic, clinical, and essential tremor-related characteristics.

**Table 1:** Baseline and essential tremor-related characteristics of participants.

Variable	Total (*n* = 88)	Group		*P*-value
		Intervention (*n* = 45)	Non-intervention (*n* = 43)	
*Demographic and medical history-related variables*				
Age (y/o)	63.5 [52.5, 70.3]	65.0 [58.0, 72.0]	59.0 [47.0, 67.0]	0.079
Sex (male)	51 (57.95)	30 (66.67)	21 (48.84)	0.130
Smoking	12 (13.64)	8 (17.78)	4 (9.30)	0.354
Comorbid conditions				
Diabetes mellites	18 (20.45)	11 (24.44)	7 (16.28)	0.431
Hypertension	28 (31.82)	13 (28.89)	15 (34.88)	0.649
Dyslipidemia	32 (36.36)	20 (44.44)	12 (27.91)	0.125
Cardiovascular diseases ^†^	23 (26.14)	14 (31.11)	9 (20.93)	0.336
Cerebrovascular diseases ^‡^	4 (4.55)	1 (2.22)	3 (6.98)	0.355
Psychiatric conditions ^§^	31 (35.23)	12 (26.67)	19 (44.19)	0.118
*Essential tremor-related variables*				
Essential tremor duration (year)	5.0 [3.0, 12.8]	5.0 [3.0, 20.0]	5.0 [3.0, 10.5]	0.525
Pharmacotherapy (yes)	64 (72.73)	31 (68.89)	33 (76.74)	0.296
*Monotherapy*				
Propranolol	24 (27.27)	12 (26.67)	12 (27.91)	1.000
Primidone	18 (20.45)	8 (17.78)	10 (23.26)	0.602
Gabapentin	1 (1.14)	0 (0)	1 (2.33)	0.489
*Dual therapy*				
Propranolol, primidone	15 (17.05)	9 (20.00)	6 (13.95)	0.574
Propranolol, ASM^||^	1 (1.14)	1 (2.22)	0 (0)	1.000
Primidone, ASM^||^	2 (2.27)	0 (0)	2 (4.65)	0.236
*Triple therapy*				
Propranolol, primidone, ASM^||^	3 (3.41)	1 (2.22)	2 (4.65)	0.612
Duration of pharmacotherapy (month)	18.0 [1.5, 36.0]	24.0 [2.4, 49.5]	6.0 [1.0, 36.0]	0.268

Numerical variables are represented as mean ± standard deviation (SD) or median (interquartile range [IQR]). Categorical variables are expressed as frequency (percentage). Statistical comparisons of numerical variables between treatment groups were conducted using Independent-Samples T-tests or Mann-Whitney-U test. The relationship between categorical variables and the intervention was assessed employing the Fisher exact test. *Abbreviations*: ASM: antiseizure medication; y/o: years old.

^†^Cardiovascular diseases were defined as coronary artery disease, myocardial infarction, heart failure, atrial fibrillation, cardiac arrhythmia, and valvular heart diseases.

^‡^Cerebrovascular diseases were defined as cerebral infarction, intracerebral hemorrhage, and subarachnoid hemorrhage.

^§^Psychiatric conditions included major depressive disorder, bipolar disorder, and anxiety disorders diagnosed by a psychiatrist. People with psychiatric conditions were included only if their conditions were stable and they were under medical treatment and supervision.

^||^Antiseizure medications included gabapentin, topiramate, or levetiracetam.

### Temporal evolution of treatment-induced changes in efficacy outcomes

Table 2 presents the temporal changes in the outcomes over time by groups. Supplementary Figure S1 visually presents these changes between pre-intervention and subsequent time points for each group, providing a clear understanding of the changes over time. Supplementary Figures S2 and S3 show the changes in each of the evaluated TETRAS and BF-ADL task performance over time across the groups.

**Table 2:** Comparison of efficacy outcomes at different follow-up time points among groups.

Variables	Group	Baseline	Post-intervention time points				*P* ^†^
		Pre-int	Immediately	30 min	60 min	90 min	
*Tremor amplitude* (m/s^2^)^**†**^	Int	1420.94 ± 239.95	767.74 ± 616.77	859.34 ± 675.90	952.76 ± 651.36	948.80 ± 572.91	< 0.001
	Non-int	1334.40 ± 327.05	1227.64 ± 475.78	1159.36 ± 486.84	1228.87 ± 451.85	1207.13 ± 443.58	0.034
*Total TETRAS*	Int	11.11 ± 5.53	8.11 ± 5.32	8.60 ± 5.79	8.64 ± 5.87	8.42 ± 5.91	< 0.001
	Non-int	9.86 ± 5.20	8.02 ± 5.42	8.23 ± 5.55	8.07 ± 5.44	7.88 ± 5.60	< 0.001
Forward postural	Int	1.80 ± 0.82	1.20 ± 0.69	1.31 ± 0.70	1.24 ± 0.88	1.29 ± 0.82	< 0.001
	Non-int	1.74 ± 0.76	1.16 ± 0.92	1.35 ± 0.78	1.35 ± 0.78	1.23 ± 0.81	0.002
Lateral postural	Int	1.76 ± 0.88	1.27 ± 0.89	1.27 ± 0.92	1.42 ± 0.94	1.36 ± 0.88	0.019
	Non-int	1.67 ± 0.84	1.40 ± 0.82	1.42 ± 0.88	1.35 ± 0.92	1.33 ± 0.97	< 0.001
Kinetic	Int	1.69 ± 1.10	1.20 ± 1.01	1.33 ± 1.13	1.33 ± 1.09	1.20 ± 1.16	< 0.001
	Non-int	1.16 ± 1.15	0.98 ± 0.94	0.91 ± 1.04	0.88 ± 1.01	0.79 ± 1.04	0.002
Spiral drawing	Int	2.11 ± 1.17	1.49 ± 1.20	1.69 ± 1.26	1.60 ± 1.27	1.56 ± 1.25	< 0.001
	Non-int	1.93 ± 1.14	1.53 ± 1.24	1.58 ± 1.28	1.56 ± 1.22	1.51 ± 1.24	< 0.001
Handwriting	Int	1.96 ± 1.22	1.69 ± 1.35	1.56 ± 1.32	1.62 ± 1.34	1.58 ± 1.31	0.005
	Non-int	1.86 ± 1.25	1.58 ± 1.35	1.56 ± 1.37	1.58 ± 1.22	1.60 ± 1.35	0.062
Dot approximation	Int	1.80 ± 1.38	1.27 ± 1.27	1.44 ± 1.36	1.42 ± 1.25	1.44 ± 1.32	0.150
	Non-int	1.49 ± 1.40	1.37 ± 1.45	1.42 ± 1.42	1.35 ± 1.34	1.42 ± 1.26	0.488
*Total BF-ADL*	Int	15.27 ± 5.89	12.58 ± 5.26	11.66 ± 5.23	12.02 ± 5.60	12.16 ± 5.86	< 0.001
	Non-int	14.02 ± 4.95	12.51 ± 4.27	12.28 ± 5.38	11.35 ± 4.52	11.42 ± 4.53	< 0.001
Spoon using	Int	2.49 ± 1.01	2.02 ± 0.97	1.87 ± 0.97	1.89 ± 1.03	1.96 ± 1.04	< 0.001
	Non-int	2.35 ± 1.00	2.00 ± 0.98	1.74 ± 1.00	1.74 ± 0.95	1.70 ± 0.89	< 0.001
Cup holding	Int	1.96 ± 0.93	1.51 ± 0.84	1.56 ± 0.87	1.44 ± 0.79	1.42 ± 0.84	< 0.001
	Non-int	1.81 ± 0.91	1.72 ± 0.91	1.58 ± 0.91	1.63 ± 0.93	1.56 ± 0.98	0.025
Milk pouring	Int	2.13 ± 1.06	1.82 ± 1.01	1.64 ± 0.98	1.62 ± 0.98	1.62 ± 1.01	< 0.001
	Non-int	1.74 ± 0.93	1.70 ± 0.94	1.60 ± 0.96	1.53 ± 0.88	1.51 ± 0.83	0.030
Phone dialing	Int	1.73 ± 0.92	1.33 ± 0.71	1.38 ± 0.75	1.24 ± 0.53	1.24 ± 0.57	< 0.001
	Non-int	1.56 ± 0.91	1.23 ± 0.68	1.21 ± 0.60	1.26 ± 0.62	1.21 ± 0.56	0.027
Coin picking up	Int	1.60 ± 1.05	1.29 ± 0.63	1.44 ± 0.94	1.40 ± 0.86	1.42 ± 0.94	0.247
	Non-int	1.42 ± 0.76	1.12 ± 0.39	1.14 ± 0.52	1.14 ± 0.52	1.14 ± 0.52	0.005
Plugging	Int	1.51 ± 0.79	1.27 ± 0.62	1.24 ± 0.53	1.27 ± 0.62	1.33 ± 0.74	0.268
	Non-int	1.53 ± 0.74	1.44 ± 0.70	1.23 ± 0.61	1.23 ± 0.61	1.33 ± 0.72	0.011
Door unlocking	Int	1.64 ± 0.68	1.44 ± 0.73	1.47 ± 0.79	1.33 ± 0.67	1.38 ± 0.68	0.008
	Non-int	1.58 ± 0.79	1.44 ± 0.77	1.28 ± 0.63	1.21 ± 0.60	1.30 ± 0.67	0.002
Letter writing	Int	2.20 ± 1.01	1.89 ± 0.94	1.87 ± 1.01	1.82 ± 0.96	1.78 ± 0.93	< 0.001
	Non-int	2.02 ± 0.94	1.86 ± 0.97	1.72 ± 0.88	1.60 ± 0.88	1.67 ± 0.84	< 0.001
*CGI-I*	Int	----	2.53 ± 0.89	2.53 ± 0.99	2.49 ± 1.10	2.51 ± 1.14	0.851
	Non-int	----	3.02 ± 0.91	2.93 ± 1.12	2.91 ± 1.11	2.72 ± 1.14	0.020

Note: Generalized Estimating Equations (GEE) were used to assess the temporal changes in outcomes across the follow-up time points. Symbols † represent the p-values derived from GEE analyses, indicating the significance of changes in outcomes from pre-intervention to 90 min post-intervention, respectively. *Abbreviations*: BF-ADL: Bain and Findley Activities of Daily Living; CGI-I: Clinical Global Impression-Improvement; Int: intervention group; Non-int: non-intervention group; TETRAS: Essential Tremor Rating Assessment Scale; min: minutes.

^†^ The sensors of the accelerometer were 16-bit devices and the sensitivity was between − 2G and + 2G. G = 9.8 m/s^2^.

*Tremor amplitude* (m/s^2^): Both groups experienced significant changes in tremor amplitude over time (*p* < 0.001 for the intervention, *p* = 0.034 for the non-intervention group). However, compared to the baseline, the intervention group displayed significantly reduced amplitudes at all follow-up points, while the non-intervention group only showed a reduction at 30 min (Table 2 and Figure S1A).

*TETRAS scores*: Total TETRAS scores significantly improved over time in both groups (*p* < 0.001) (Table 2 and Figure S1B). Task assessments revealed improvements in all evaluated tasks in both groups except dot approximation.

*BF-ADL scores*: Both the intervention and non-intervention groups demonstrated significant improvements in total BF-ADL scores within the first 90 min (*p* < 0.001 for both). However, both groups showed significant changes over the extended 24-hour period (*p* < 0.046 for the intervention, *p* < 0.001 for the non-intervention group) (Table 2, Figure S1C, and Supplementary Table S2). Task assessments showed improvements in all evaluated tasks for both groups within the 90-minute follow-up, except for coin picking and plugging. These tasks only significantly improved in the non-intervention group (coin picking: *p* = 0.005, plugging: *p* = 0.011), with no significant improvement observed in the intervention group (coin picking: *p* = 0.247, plugging: *p* = 0.268).

*CGI-I scores*: The non-intervention group displayed significant changes in CGI-I scores during the first 90 min (*p* = 0.020). Such change was not observed in the intervention group (*p* = 0.851) (Table 2, Figure S1D, Supplementary Table S2).

### Comparative analysis of study groups on efficacy outcomes across study time points

**Fig. 3:**
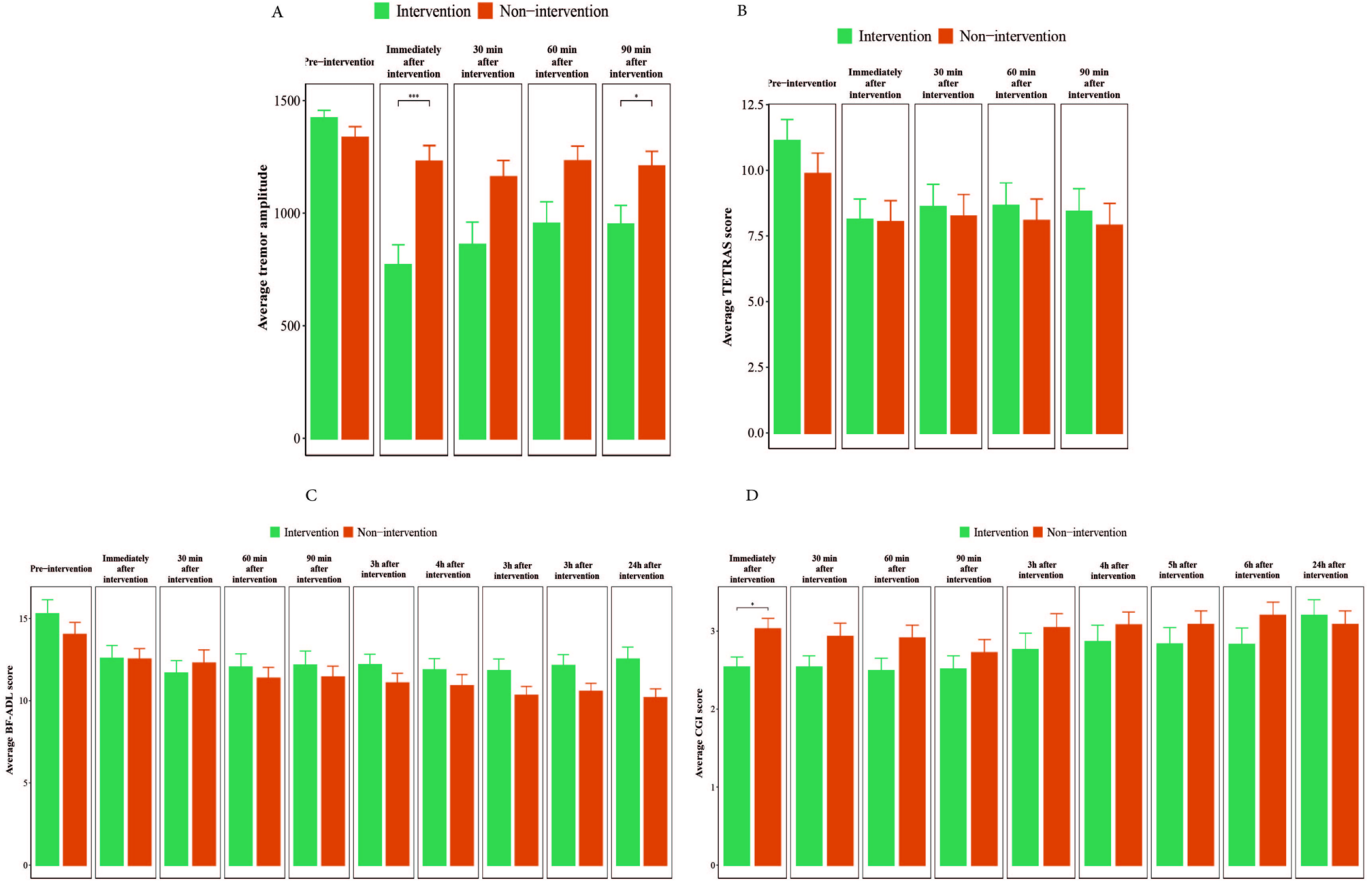
Comparing average outcomes between intervention and non-intervention groups within study time points. *BF-ADL* Bain and Findley Activities of Daily Living, *CGI-I* Clinical Global Impression-Improvement, *TETRAS* Tremor Research Group Essential Tremor Rating Assessment Scale, *min* minute(s), *h* hour(s). Accelerometer sensors were 16-bit devices and the sensitivity was between − 2G and + 2G. G = 9.8 m/s^2^. *Significant at P-value < 0.05. **significant at P-value < 0.01. ***Significant at P-value < 0.001. ****Significant at P-value < 0.0001.

Figure 3 presents bar charts comparing the mean outcomes for the intervention and non-intervention groups at each follow-up time point. Tremor amplitude showed a significant difference at “immediately after” and “90-minute” post-stimulation. During these specific intervals, the intervention group exhibited a significantly lower mean amplitude compared to the non-intervention group (Fig. 3A).

In contrast, TETRAS and BF-ADL scores displayed no statistically significant differences between the groups at any time point (Fig. 3B and C). CGI-I scores yielded similar results, except for the “immediately after intervention” time point where the intervention group exhibited a significantly better score compared to the non-intervention group (Fig. 3D). Supplementary Figures S4 and S5 compare average TETRAS and BF-ADL task performance between groups at each study time point.

### Impact of treatment groups on efficacy outcomes over time in a longitudinal manner

To determine the intervention efficacy, our main interest lies in how the intervention impacts outcomes over time, rather than simple group differences. Table 3 summarizes the results of GEE analyses (within the 90-minute post-stimulation), which examined the effects of group (intervention vs. non-intervention), time, and their interaction on the outcome measures. Supplementary Figure S6 demonstrates temporal trends in outcomes over time by treatment groups.

**Table 3:** The impact of group, time, and their interaction on efficacy outcomes (within 90 min).

Outcome	Parameter	B (95% CI)	*P*-value
*Tremor amplitude* (m/s^2^)	Intervention vs. Non-intervention	−139.36 (−294.19, 15.48)	0.078
	Time	−25.33 (−48.76, −1.91)	0.034
	[Intervention vs. Non-intervention] *Time	−51.61 (−94.60, −8.62)	0.019
*Total TETRAS*	Intervention vs. Non-intervention	0.75 (−1.40, 2.91)	0.494
	Time	−0.39 (−0.57, −0.21)	< 0.001
	[Intervention vs. Non-intervention] *Time	−0.09 (−0.37, 0.19)	0.512
Forward postural	Intervention vs. Non-intervention	0.03 (−0.26, 0.32)	0.843
	Time	−0.08 (−0.14, −0.03)	0.002
	[Intervention vs. Non-intervention] *Time	−0.01 (−0.09, 0.06)	0.723
Lateral postural	Intervention vs. Non-intervention	−0.04 (−0.36, 0.28)	0.812
	Time	−0.07 (−0.12, −0.03)	0.001
	[Intervention vs. Non-intervention] *Time	0.01 (−0.06, 0.08)	0.779
Kinetic	Intervention vs. Non-intervention	0.41 (0.00, 0.82)	0.052
	Time	−0.08 (−0.14, −0.03)	0.002
	[Intervention vs. Non-intervention] *Time	0.00 (−0.07, 0.07)	0.984
Spiral drawing	Intervention vs. Non-intervention	0.10 (−0.38, 0.58)	0.673
	Time	−0.08 (−0.13, −0.03)	0.001
	[Intervention vs. Non-intervention] *Time	−0.02 (−0.09, 0.05)	0.587
Handwriting	Intervention vs. Non-intervention	0.11 (−0.40, 0.61)	0.682
	Time	−0.05 (−0.11, 0.00)	0.062
	[Intervention vs. Non-intervention] *Time	−0.03 (−0.11, 0.04)	0.415
Dot approximation	Intervention vs. Non-intervention	0.15 (−0.41, 0.70)	0.610
	Time	−0.02 (−0.06, 0.03)	0.488
	[Intervention vs. Non-intervention] *Time	−0.04 (−0.13, 0.05)	0.384
*Total BF-ADL*	Intervention vs. Non-intervention	0.12 (−1.71, 1.94)	0.902
	Time	−0.37 (−0.57, −0.18)	< 0.001
	[Intervention vs. Non-intervention] *Time	0.19 (−0.09, 0.46)	0.183
Spoon using	Intervention vs. Non-intervention	0.07 (−0.32, 0.45)	0.738
	Time	−0.16 (−0.23, −0.09)	< 0.001
	[Intervention vs. Non-intervention] *Time	0.04 (−0.05, 0.13)	0.432
Cup holding	Intervention vs. Non-intervention	0.02 (−0.32, 0.37)	0.897
	Time	−0.06 (−0.11, −0.01)	0.025
	[Intervention vs. Non-intervention] *Time	−0.05 (−0.13, 0.02)	0.161
Milk pouring	Intervention vs. Non-intervention	0.27 (−0.12, 0.66)	0.175
	Time	−0.06 (−0.12, −0.01)	0.030
	[Intervention vs. Non-intervention] *Time	−0.06 (−0.14, 0.02)	0.127
Phone dialing	Intervention vs. Non-intervention	0.17 (−0.15, 0.49)	0.294
	Time	−0.07 (−0.13, −0.01)	0.027
	[Intervention vs. Non-intervention] *Time	−0.04 (−0.12, 0.04)	0.318
Coin picking up	Intervention vs. Non-intervention	0.18 (−0.11, 0.48)	0.228
	Time	−0.05 (−0.09, −0.02)	0.005
	[Intervention vs. Non-intervention] *Time	0.03 (−0.03, 0.09)	0.307
Plugging	Intervention vs. Non-intervention	−0.08 (−0.36, 0.19)	0.552
	Time	−0.06 (−0.11, −0.02)	0.011
	[Intervention vs. Non-intervention] *Time	0.03 (−0.05, 0.11)	0.501
Door unlocking	Intervention vs. Non-intervention	0.06 (−0.22, 0.34)	0.668
	Time	−0.08 (−0.13, −0.03)	0.002
	[Intervention vs. Non-intervention] *Time	0.02 (−0.06, 0.08)	0.679
Letter writing	Intervention vs. Non-intervention	0.13 (−0.25, 0.50)	0.511
	Time	−0.10 (−0.15, −0.05)	< 0.001
	[Intervention vs. Non-intervention] *Time	0.00 (−0.07, 0.08)	0.906
*CGI-I*	Intervention vs. Non-intervention	−0.46 (−0.90, −0.03)	0.038
	Time	0.03 (−0.05, 0.10)	0.502
	[Intervention vs. Non-intervention]*Time	0.05 (−0.06, 0.15)	0.390

The generalized estimation equation (GEE) was used to evaluate the impact of each group, time, and group*time interaction on the outcomes and tasks. *BF-ADL* Bain and Findley Activities of Daily Living, *CGI-I* Clinical Global Impression-Improvement, *TETRAS* Tremor Research Group Essential Tremor Rating Assessment Scale.

Statistically significant interactions were observed between the time and treatment group for tremor amplitude (B = −51.61, 95% CI [−94.60, −8.62], *p* = 0.019). This indicates that the intervention group experienced a significantly greater reduction in tremor amplitude over time compared to the non-intervention group.

While the intervention significantly impacted tremor amplitude over time, we did not observe a similar interaction effect for other outcomes. Supplementary Figures S7 and S8 depict temporal trends in each evaluated TETRAS and BF-ADL tasks over time by treatment groups.

### Adverse events

One participant in the non-intervention group experienced mild, transient skin irritation (redness, itching) that self-resolved within 15–20 min. No other adverse events were observed.

## Discussion

We investigated the effects of a single-session PNS on essential tremor. Objective efficacy was assessed using accelerometer-measured tremor amplitude (m/s^2^) and TETRAS task performance. Subjective efficacy included patients’ performance in BF-ADL tasks and their self-reported improvement (CGI-I). PNS induced a significantly greater reduction in tremor amplitude compared to sham procedure, with effects persisting for 90 min post-stimulation. However, no interaction effects were observed for other evaluated outcomes. No major safety concern was identified.

### Prior PNS research and its challenges in essential tremor

The introduction of the Cala Trio device in 2018 sparked a surge in research exploring median and radial nerve stimulation for the treatment of essential tremor^19^. Building on the initial promise of this approach^19–21,24–26^, our study represents the seventh in this field and the first to investigate a Middle Eastern population. Supplementary Table S3 summarizes relevant studies. Prior research includes three trials on the acute short-term effects of PNS on hand tremor (two sham-controlled^19,20^ and one single-arm^25^, as well as three single-arm studies on the device’s long-term use^21,24,26^. Due to the difficulty of duplicating the sensation induced by stimulation while maintaining blindness throughout continuous device use at home, these latter studies lacked a control group^21,24,26^. Notably, while previous sham-controlled studies provided valuable insights, they only compared effects at baseline and immediately post-stimulation, overlooking durability^19,20^. Only one study (*N* = 15) investigated durability but lacked a control group^25^. They reported persistent improvements in tremor power in over 70% of patients (10 of 15 participants) for at least 60 min beyond stimulation^25^. Our study addresses these gaps by evaluating patients at multiple follow-up points, including 90 min after stimulation for TETRAS and accelerometer-measured tremor amplitude, and 24 h for BF-ADL and CGI-I. Our results indicated a significantly greater tremor amplitude reduction in the intervention group than in the sham group within the 90-minute post-stimulation period.

The first double-arm study (2018) with a limited sample size (*N* = 23) showed significant improvement in the intervention group’s TETRAS spiral drawing test compared to the baseline. Such improvement was not observed in the sham group^19^. The second double-arm study (2019), with a larger sample size (40 intervention, 37 sham), examined four TETRAS tasks, seven tasks from BF-ADL, and CGI-I^20^. Among the evaluated TETRAS tasks (spiral drawing, forward postural, lateral postural, and kinetic), only the forward postural task demonstrated a substantially greater improvement in the intervention group than the sham group^20^. Regarding BF-ADL tasks, the intervention group demonstrated significantly greater improvements in four of seven tasks compared to the sham group (cup holding, phone dialing, coin pick-up, door unlocking)^20^. Notably, both groups improved in some BF-ADL tasks compared to baseline, highlighting the sham effect’s influence, particularly in research including neurological devices^20^. Self-reported improvement (CGI-I) was also significantly better in the intervention group^20^. Potential explanations for the discrepancies between our findings (non-significant interactions for efficacy outcomes except tremor amplitude) and those of the previous studies include differing TETRAS and BF-ADL thresholds for participant inclusion, resulting in different tremor severities. Unlike the prior studies, our research did not exclude patients with mild tremors, aiming to better reflect real-world scenarios. Additionally, variations in the study population, statistical methodologies, and the potential need for population-specific tremor severity scale validation may contribute to the observed differences.

A subsequent prospective study without a control group (205 participants, 3 months) showed significant improvements in TETRAS, BF-ADL, and tremor power following PNS compared to baseline^21^. While this study reported significant improvements in all investigated TETRAS and BF-ADL tasks compared to baseline, it’s important to consider the limitations of studies without control groups^21^. In our study, while both the intervention and sham groups showed a trend of improvement over time for most tasks, GEE analyses did not reveal significant interactions between time and the treatment group. In other words, despite receiving no active stimulation, participants in the sham group demonstrated improvements in several tasks. This phenomenon may be attributed to a combination of factors commonly observed in device-based clinical trials. First, unconscious expectancy effects may arise simply from participating in a structured study and using a therapeutic-looking device, even when participants suspect they are in the sham group. Second, practice or learning effects are likely contributors, as repeated exposure to TETRAS and BF-ADL tasks can lead to improved performance regardless of intervention. Third, the Hawthorne effect may have played a role, with participants modifying their behavior simply because they were being observed and assessed^38^. Fourth, the psychological impact of hope or perceived care—generated by clinical attention and the use of a wearable device—can enhance motivation and performance. Finally, non-sensory cues from the device itself—such as pressure from the wristband, or visual or tactile awareness—might influence sensorimotor function independently of neural stimulation. Importantly, while such effects may bias subjective or task-based outcomes, they are far less likely to influence objective physiological measures such as tremor amplitude quantified by accelerometer data, which provides a high-resolution and observer-independent assessment of motor function.

Two retrospective post-market surveillance studies (2022, 2023) measured the effects of chronic PNS use over 90 days^24,26^. Interestingly, these studies suggested a greater reduction in tremor for those with initially more severe tremors^24,26^. Notably, whether PNS can be used alone or as an adjunct to medication requires further investigation. However, Isaacson et al.‘s study suggested similar device efficacy regardless of medication use^21^. Lu’s study reported that around 60% of patients reduced their medication or planned to discuss it with their physicians^24^.

### Mechanisms of reducing essential tremor during PNS

Essential tremor is thought to be linked to disruptions in the cerebellum and cerebellothalamocortical circuits, potentially due to the degeneration of Purkinje fibers^39–41^. Electrical stimulation through PNS may improve tremor by influencing an oscillating network within these circuits, ultimately impacting motor function^42^. While the exact mechanism remains unclear, several hypotheses exist. One theory suggests that stimulating the median nerve, which carries both sensory and motor signals, activates both pathways. This activation could cause muscle contractions and interfere with abnormal sensorimotor activity, leading to reduced tremor^43,44^. However, in our study, the stimulation amplitude remained below the motor threshold to avoid muscle contractions. Another hypothesis proposes that the firing pattern of median nerve stimulation disrupts abnormal thalamic activity, specifically in the VIM of the thalamus^22,23,45^. Finally, PNS might indirectly affect the cerebellum through its projections to higher brain regions within the cerebellothalamocortical circuits. This altered cerebellar output could ultimately influence motor function and reduce tremor^46^.

### Limitations, strengths, and future directions

The study limitations and suggestions for future studies are outlined below. The most significant challenges—such as the placebo effect, potential unblinding risk, and single-session design—were inherent to the study design and could not be fully mitigated.

- *Single-center recruitment*: The participants were recruited from a single academic hospital, limiting the generalizability of the findings to other populations.
- *Concurrent medication use*: Participants were allowed to continue their tremor medication, reflecting real-world scenarios. However, this makes it challenging to isolate the specific effects of PNS from potential interactions with medications. A washout period for medications could be considered in future studies to isolate the effects of PNS.
- *Sham stimulation challenges*: A major limitation is the difficulty of achieving true sham stimulation. Unlike drug trials where an inert placebo can be identical in appearance, device-based shams—especially those attempting to simulate stimulation without any neural effect—present unique methodological challenges. Although identical devices and procedures were used to maintain blinding, participants in the sham group may have correctly inferred their allocation due to the absence of sensory feedback such as tingling or paresthesia. This potential unblinding could introduce expectancy biases, potentially diminishing placebo effects in the sham group or amplifying perceived benefits in the intervention group. However, it is noteworthy that in our study, both groups showed comparable trends of improvement across several TETRAS and BF-ADL tasks, and no significant differences were found between the groups. If participants had recognized their group allocation, we might have expected a greater benefit in the intervention group due to positive expectancy or poorer performance in the sham group due to perceived inefficacy. The absence of such group differences may suggest that participants did not strongly or uniformly guess their allocation, and that expectancy bias may have been limited. Moreover, while awareness of group allocation may bias self-reported or task-based outcomes, it is less likely to directly affect objective physiological measures such as accelerometer-measured tremor amplitude. To improve blinding, future studies might consider using low-level sub-sensory stimulation or intermittent non-functional pulses that mimic the tactile sensation of active stimulation without inducing physiological effects. However, this is challenging in PNS trials that typically require suprathreshold stimulation. Including post-intervention blinding assessments could also help evaluate the extent and impact of potential unblinding. Additionally, sham devices may incorporate non-sensory cues such as subtle lights, vibrations, or sounds to further mimic the active device and reduce the likelihood of group detection.
- *Assessment-related expectancy bias*: While TETRAS operators were blinded to the treatment groups, the logistics of the follow-up assessments at specified intervals inherently reveal the timing to the operators, which could potentially create an expectation bias. Moreover, while the TETRAS scale is considered objective, its application is inherently subject to operator judgment. However, the expertise of our TETRAS assessors, both specialists in movement disorders with extensive experience in the scoring system, helped to mitigate this potential bias.
- *Single-session design*: Our study, while offering the longest post-stimulation follow-up in a “single-session” PNS trial for essential tremor, was constrained by its single-session design. We believe a multi-session approach might have provided more statistically significant and therapeutically beneficial results. Nevertheless, practical obstacles significantly impacted this choice. Participants often faced long travel distances, and each 90-minute session—involving five repetitions of 14 motor tasks and accelerometry—was inherently demanding, raising concerns about feasibility and potential dropout rates. Importantly, prior multi-session PNS studies typically lacked sham-control groups, instead employing continuous at-home device use, making them vulnerable to placebo effects and biased interpretations. Moving forward, studies should strive to integrate sham-controlled designs with multi-session protocols and longer intra-session follow-ups to thoroughly evaluate both the immediate and cumulative therapeutic benefits of PNS.

Despite these limitations, this study offers several strengths. It is the first to investigate PNS for essential tremor in a Middle Eastern population, a previously unexplored demographic. The longitudinal design with multiple follow-up assessments provides valuable insights into the durability of potential treatment benefits. Finally, the randomized study design enhances the study’s rigor by mitigating the placebo effect and improving the reliability of findings.

## Conclusion

In conclusion, while both intervention and sham groups demonstrated reductions in tremor amplitude over the study period, the intervention group experienced a significantly greater decrease, providing evidence for the efficacy of PNS in mitigating tremor amplitude. Despite this, there were no significant differences in the changes in TETRAS, BF-ADL, and CGI-I scores over time between the groups. This might be attributed to a strong placebo effect, single-session design, or the necessity to validate these questionnaires for each specific language and population.

## Supplementary Information

Below is the link to the electronic supplementary material.

Supplementary Material 1

## Data Availability

Data supporting the conclusions of this article will be made available by the corresponding authors (Prof. Mohammad Hossein Harirchian and Dr. Melika Jameie) upon reasonable request, without undue reservation. Contact information: harirchn@hotmail.com; Jameiemelika@gmail.com.
