# Supplementary material for: Short-term efficacy of peripheral nerve stimulation for essential tremor in a randomized double-blind controlled trial
Source: Sci Rep. 2025 Aug 6;15:28713. doi: 10.1038/s41598-025-13487-1 (PMC12328561; doi:10.1038/s41598-025-13487-1)
Supplement: Supplementary file 1 — Supplementary Material 1 [file 41598_2025_13487_MOESM1_ESM.pdf]

Supplementary Materials

Short-term efficacy of peripheral nerve stimulation for essential tremor in a randomized double-blind controlled trial

Reza Samiee¶; MD [1], Melika Jameie¶*; MD [1], Masoud Rahmati¶; MSc [2], Mehdi Azizmohammad Looha; MSc [3], Sheida Mobader; MD [1], Abbas Tafakhori; MD [1], Payam Sarraf; MD [1], Hamed Amirifard; MD [1], Sakineh Ranji Burachaloo; MD [1], Mojdeh Ghabaee; MD [1], Mobina Amanollahi; MD [4], Zohreh Tajabadi; MD [5], Mohammad Hossein Harirchian; MD [1]*

1. Iranian Center of Neurological Research, Neuroscience Institute, Tehran University of Medical Sciences, Tehran, Iran.
2. Pishgaman Rah Salamat Pars, Tehran, Iran.
3. Basic and Molecular Epidemiology of Gastrointestinal Disorders Research Center, Research Institute for Gastroenterology and Liver Diseases, Shahid Beheshti University of Medical Sciences, Tehran, Iran.
4. School of Medicine, Tehran University of Medical Sciences, Tehran, Iran.
5. Digestive Disease Research Institute, Tehran University of Medical Sciences, Tehran, Iran.

¶ Reza Samiee, Melika Jameie, and Masoud Rahmati contributed equally to this manuscript and share the first authorship.

* Mohammad Hossein Harirchian and Melika Jameie are the co-corresponding authors.

Table of Contents

**Content**		**Page**
**Table S1**	TRG essential tremor rating assessment scale (TETRAS©) V 3.1	2
**Figure S1**	Changes in task performance over time for evaluated TETARS tasks across the groups	3
**Figure S2**	Changes in task performance over time for evaluated BF-ADL tasks across the groups	4
**Figure S3**	Comparing average TETRAS task performance between groups within study time points	5
**Figure S4**	Comparing average BF-ADL task performance between groups within study time points	6
**Figure S5**	Temporal trends in each evaluated TETRAS task over time by treatment groups	7
**Figure S6**	Temporal trends in each evaluated BF-ADL task over time by treatment groups	8
**Figure S7**	Temporal trends in each evaluated TETRAS task over time by treatment groups	9
**Figure S8**	Temporal trends in each evaluated BF-ADL task over time by treatment groups	10
**Table S2**	Comparison of efficacy outcomes at different follow-up time points among groups	11
**Table S3**	Relevant studies investigating median and radial nerve stimulation for tremor	12-14
**References**		15

Table S1. TRG essential tremor rating assessment scale (TETRAS©) V 3.1

Task	Explanation
**Forward outstretched postural tremor**	Subjects should bring their arms forward, slightly lateral to midline, and parallel to the ground for 5 seconds. The wrist should also be straight and the fingers abducted so that they do not touch each other.
**Lateral "wing beating" postural tremor**	Subjects will abduct their arms parallel to the ground and flex the elbows so that the two hands do not quite touch each other and are at the level of the nose. The fingers are abducted so that they do not touch each other. The posture should be held for 20 seconds.
**Kinetic tremor (finger-nose-finger testing)**	Subjects extend only their index finger. They then touch a set object or the examiner's finger located to the full extent of their reach, which is located at the same height (parallel to the ground) and slightly lateral to the midline. Subjects then touch their own nose (or chin if the tremor is severe) and repeat this back and forth three times. Only the position along the trajectory of greatest tremor amplitude is assessed. This will typically be either at the nose or at the point of full limb extension.
**Archimedes spirals**	Demonstrate how to draw Archimedes spiral that approximately fills ¼ of an unlined page of standard (letter) paper. The lines of the spiral should be approximately 1.3 cm (0.5 inch) apart. Then ask the subject to copy the spiral. Test and score each hand separately. Use a ballpoint pen. The pen should be held such that no part of the limb touches the table. Secure the paper on the table in a location that is suitable for the patient's style of drawing. Score the tremor in the spiral, not the movement of the limb.
**Handwriting**	Have the patient write the standard sentence "This is a sample of my best handwriting" using the dominant hand only. Patients must write cursively (i.e., no printing). They cannot hold or stabilize their hand with the other hand. Use a ballpoint pen. Secure the paper on the table in a location that is suitable for the patient's style of writing. Score the tremor in the writing, not the movement of the limb.
**Dot approximation task**	The examiner makes a dot or X and instructs the subject to hold the tip of the pen "as close as possible to the dot (or center of an X) without touching it, (ideally approximately 1 mm) for 10 seconds ". Each hand is scored separately.

Figure S1. Contrasting average outcomes between pre-intervention and subsequent time points within study groups

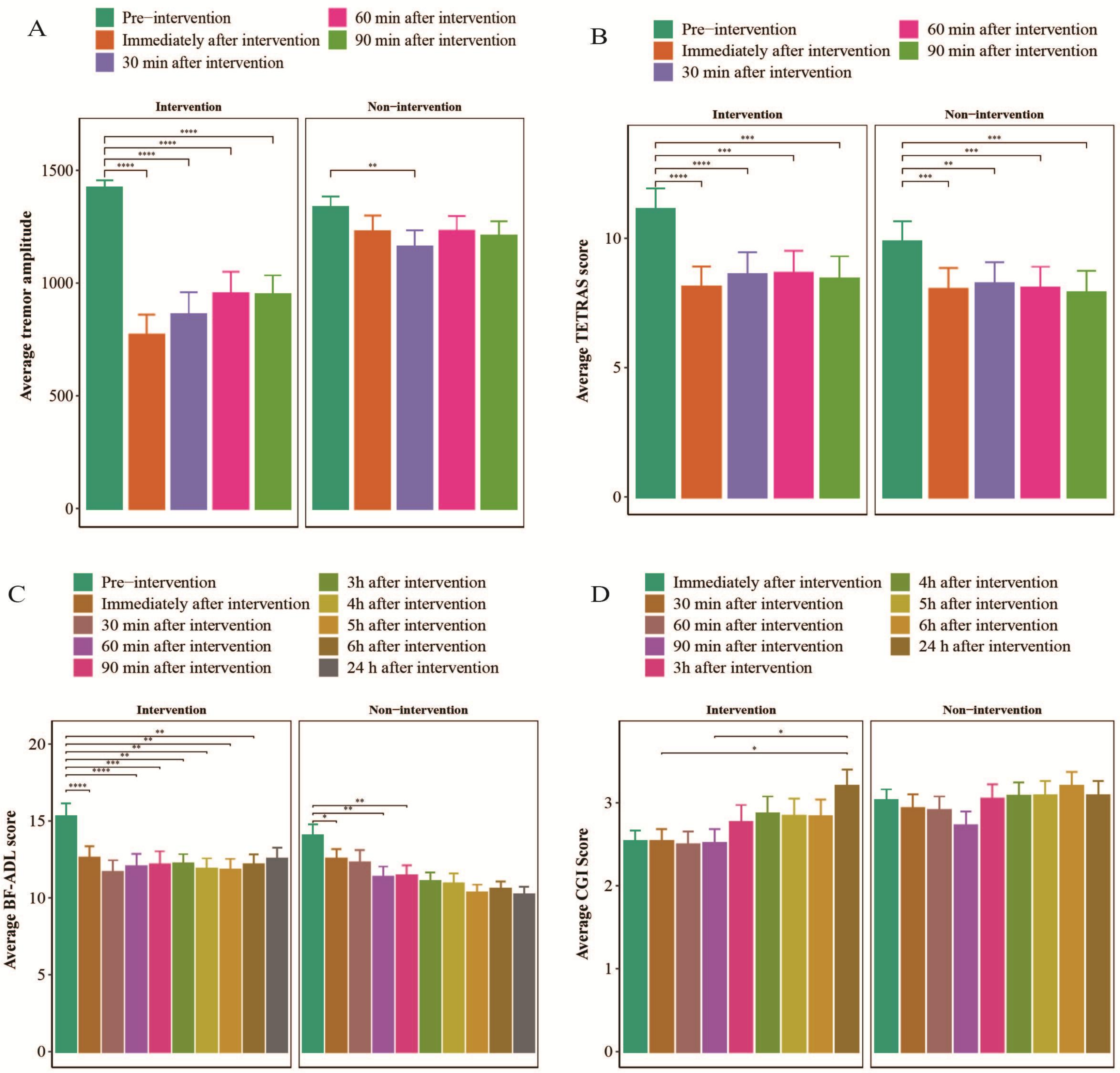

Abbreviations: BF-ADL: Bain and Findley Activities of Daily Living; CGI-I: Clinical Global Impression-Improvement; TETRAS: Tremor Research Group Essential Tremor Rating Assessment Scale; min: minute(s); h: hour(s).

Note: Accelerometer sensors were 16-bit devices and the sensitivity was between -2G and +2G. G = 9.8 m/s^2

* Significant at P-value < 0.05

** Significant at P-value < 0.01

*** Significant at P-value < 0.001

Figure S2. Changes in task performance over time for evaluated TETARS tasks across the groups

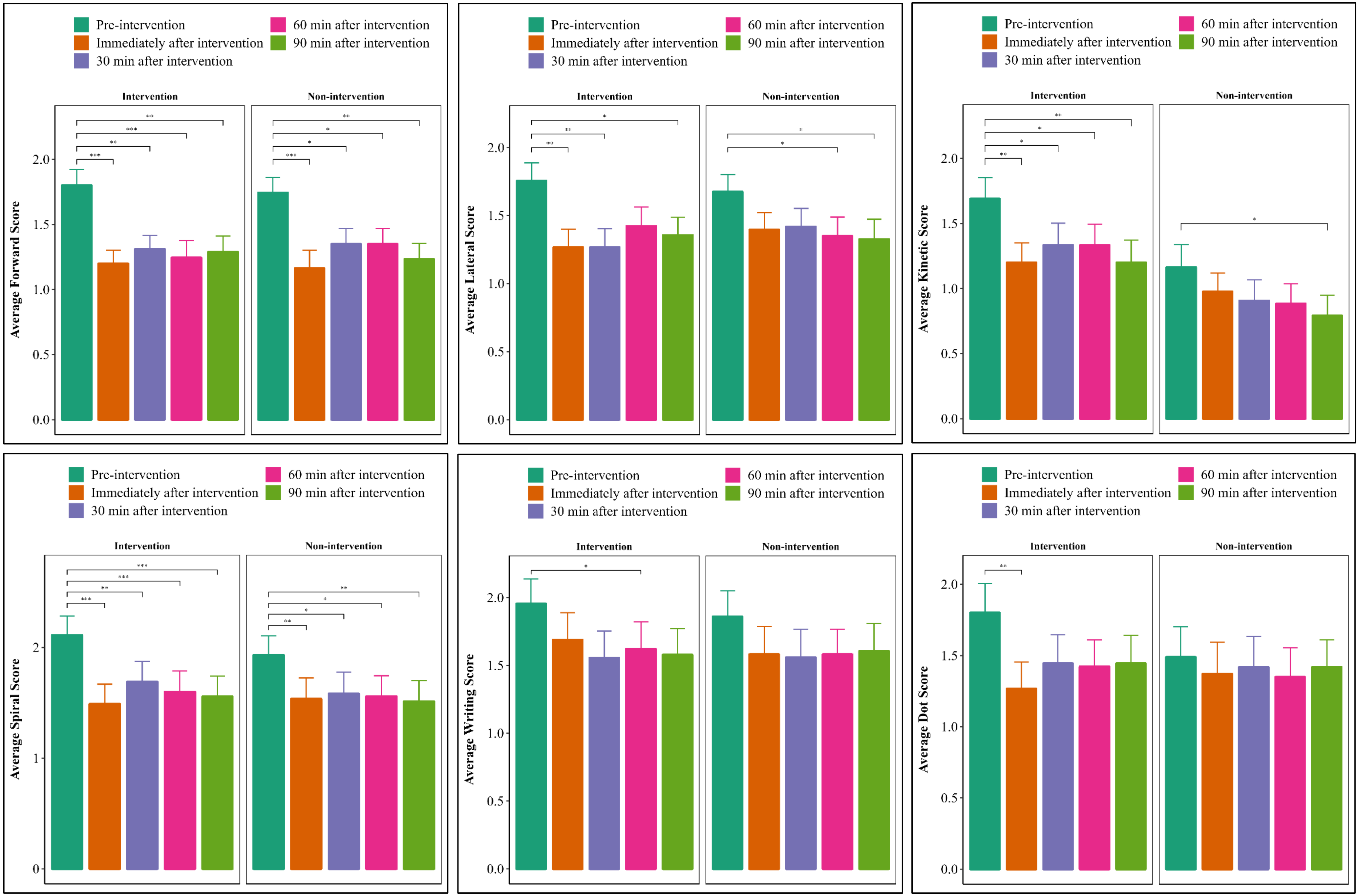

Figure S3. Changes in task performance over time for evaluated BF-ADL tasks across the groups

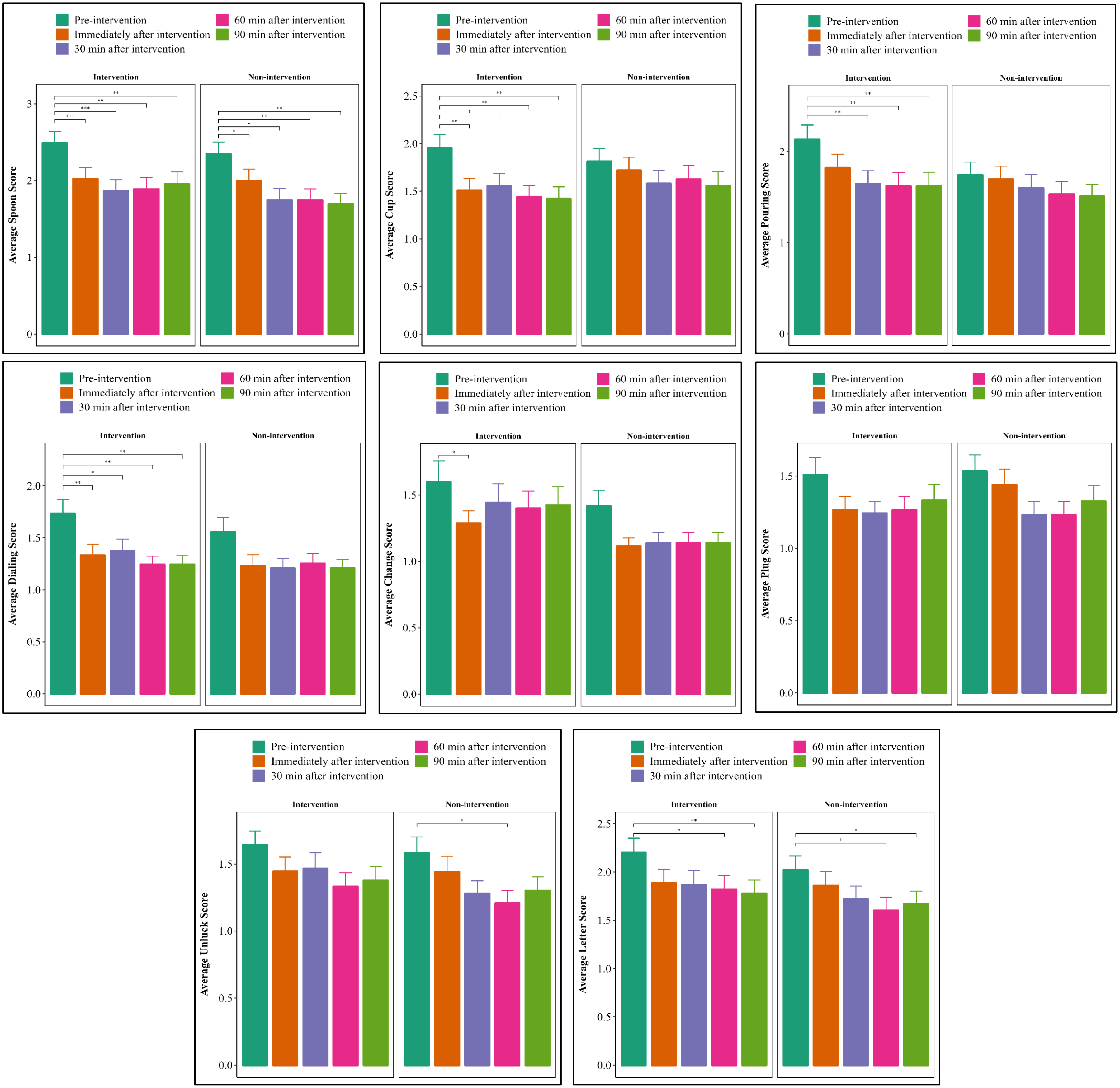

Figure S4. Comparing average TETRAS task performance between groups within study time points

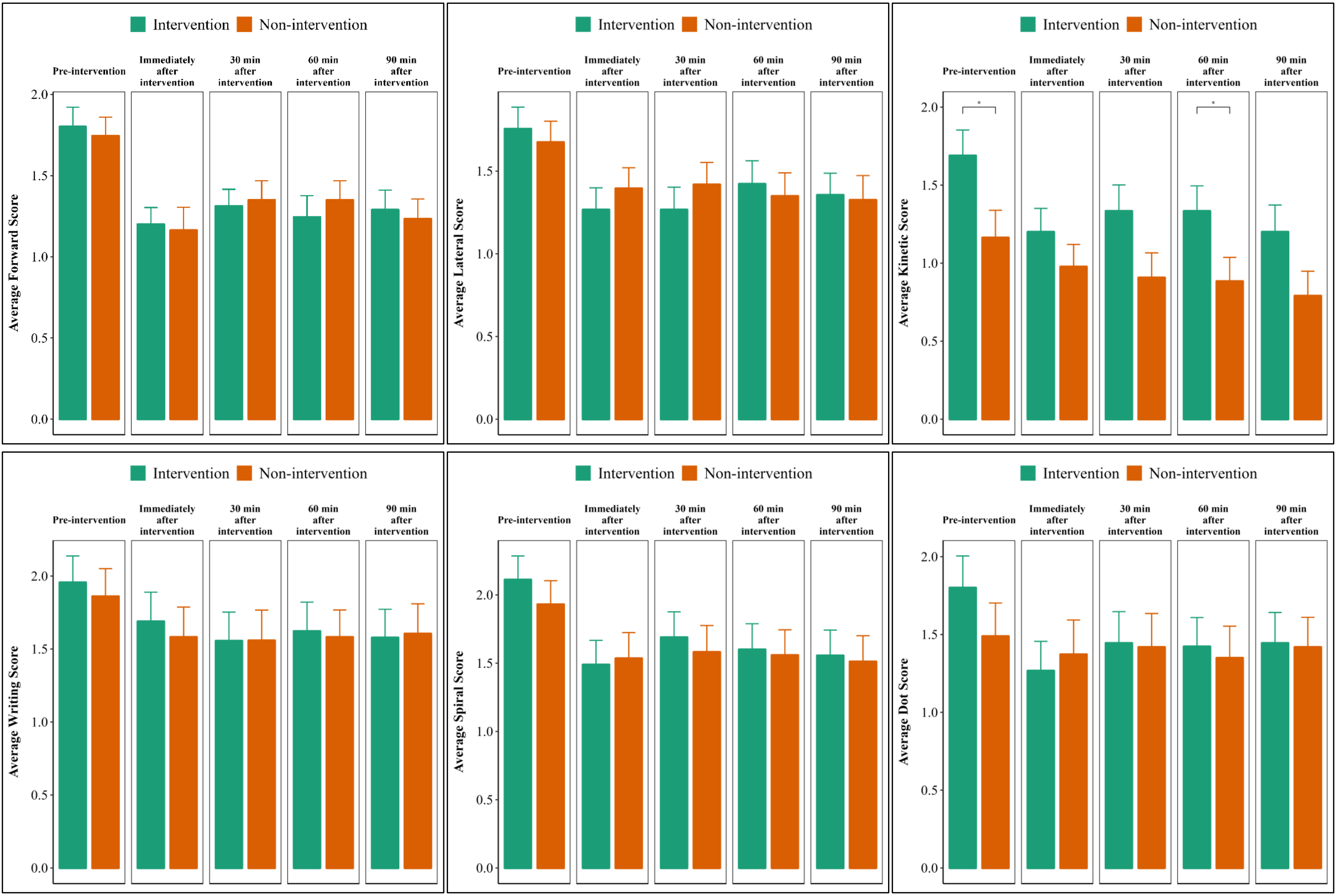

Figure S5. Comparing average BF-ADL task performance between groups within study time points

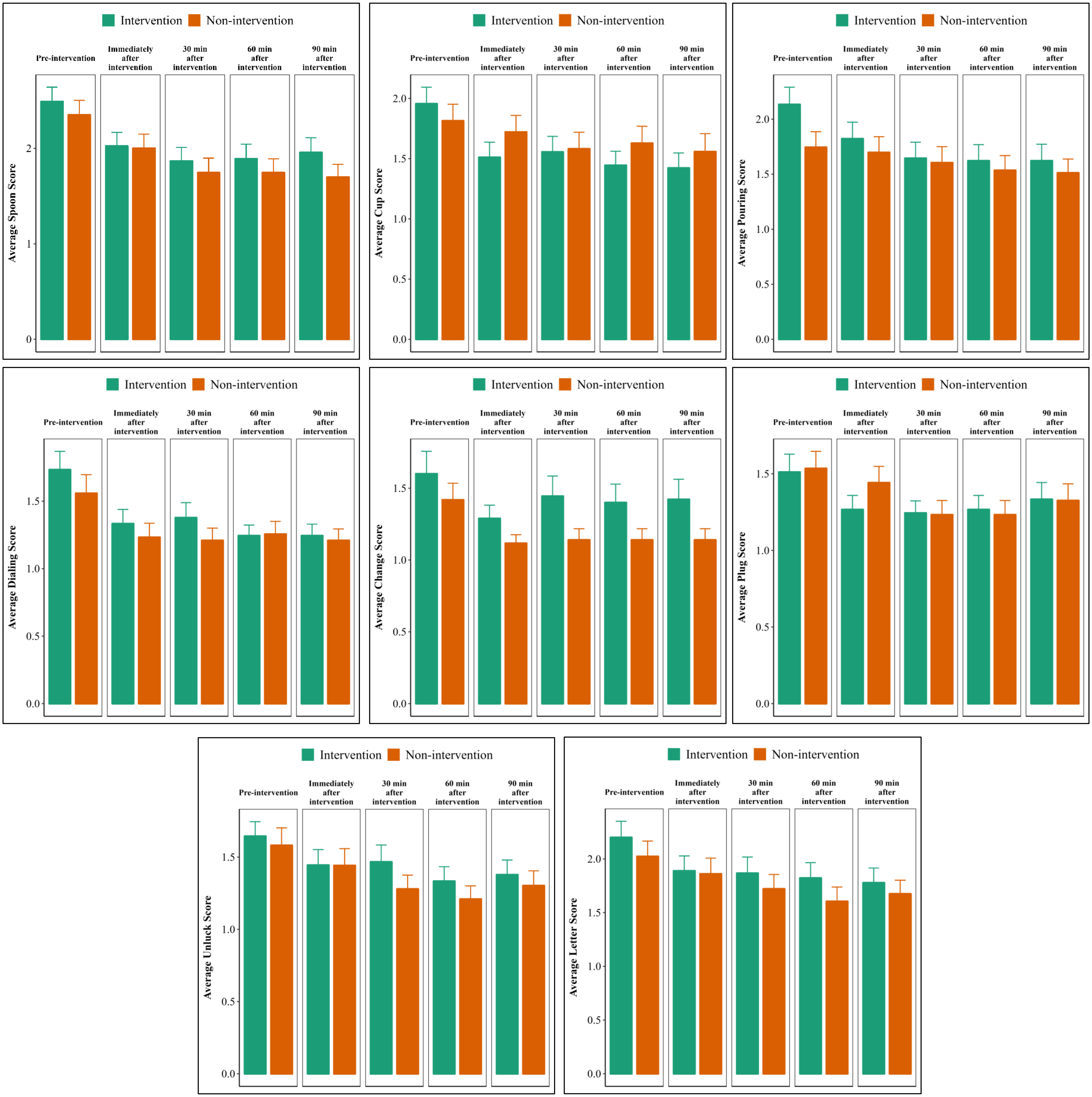

Figure S6. Temporal trends in outcomes over time by treatment groups

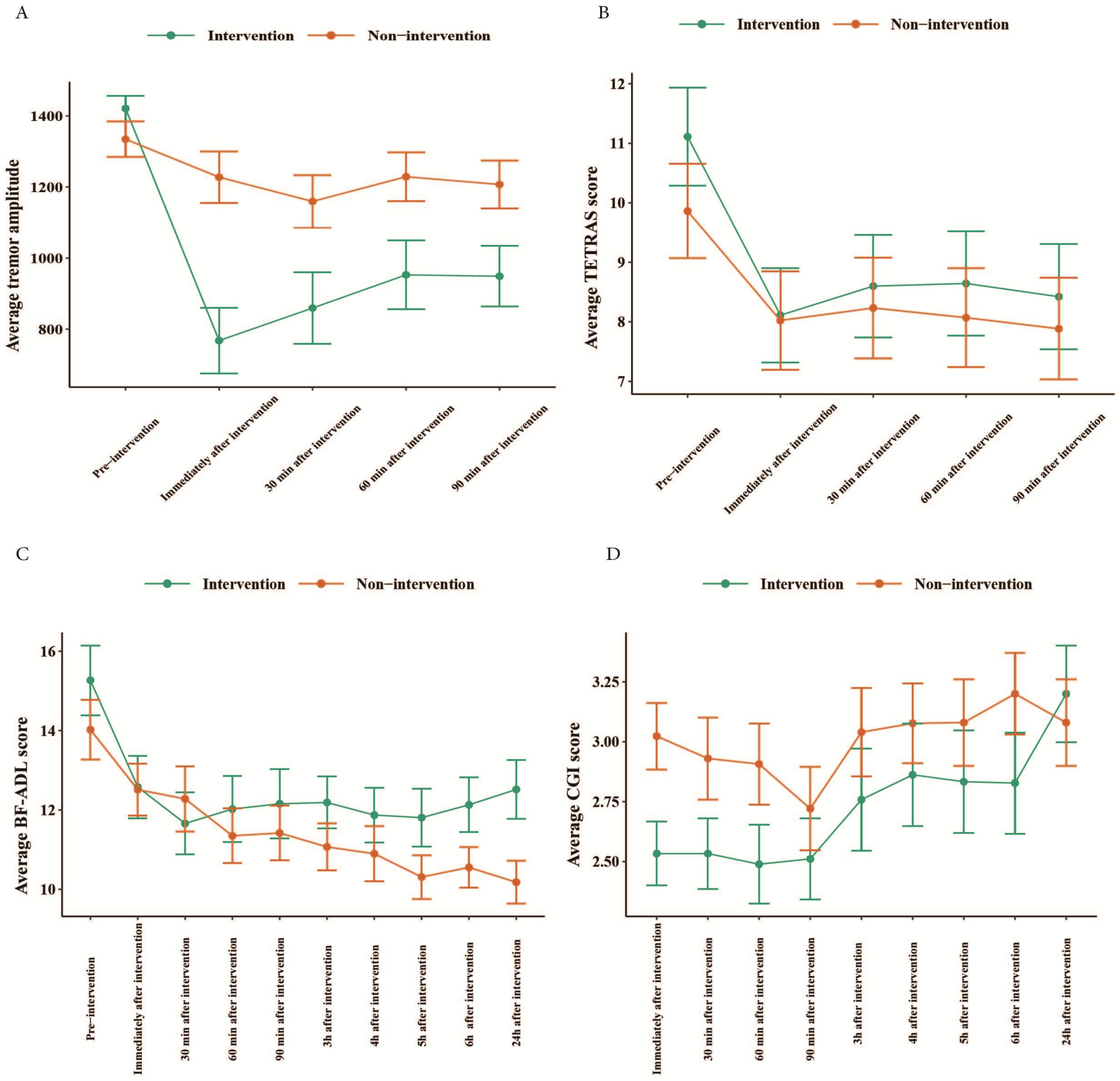

Abbreviations: BF-ADL: Bain and Findley Activities of Daily Living; CGI-I: Clinical Global Impression-Improvement; TETRAS: Tremor Research Group Essential Tremor Rating Assessment Scale; min: minute(s); h: hour(s).

Note: Accelerometer sensors were 16-bit devices and the sensitivity was between -2G and +2G. G = 9.8 m/s^2

Figure S7. Temporal trends in each evaluated TETRAS task over time by treatment groups

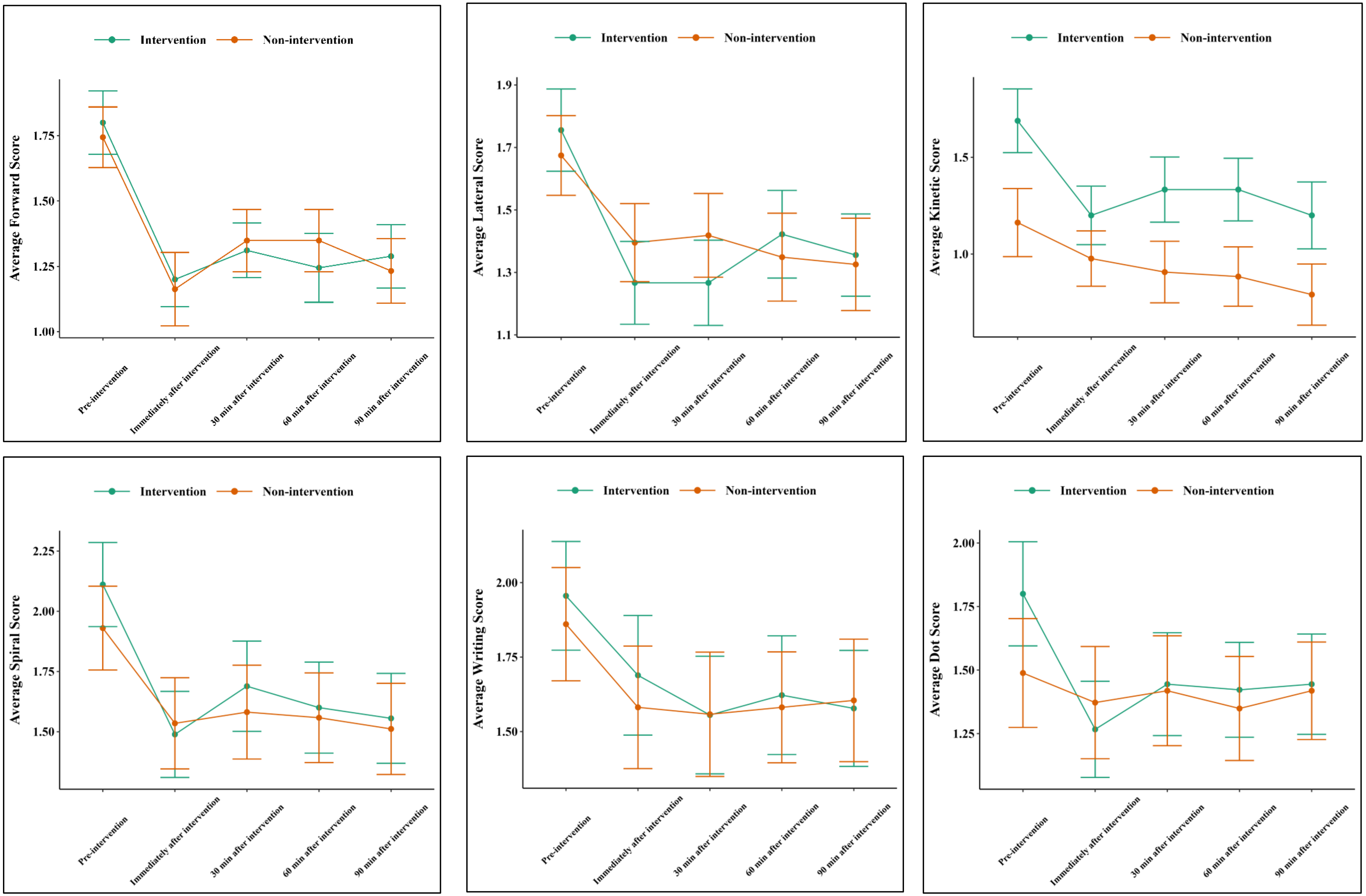

Figure S8. Temporal trends in each evaluated BF-ADL task over time by treatment groups

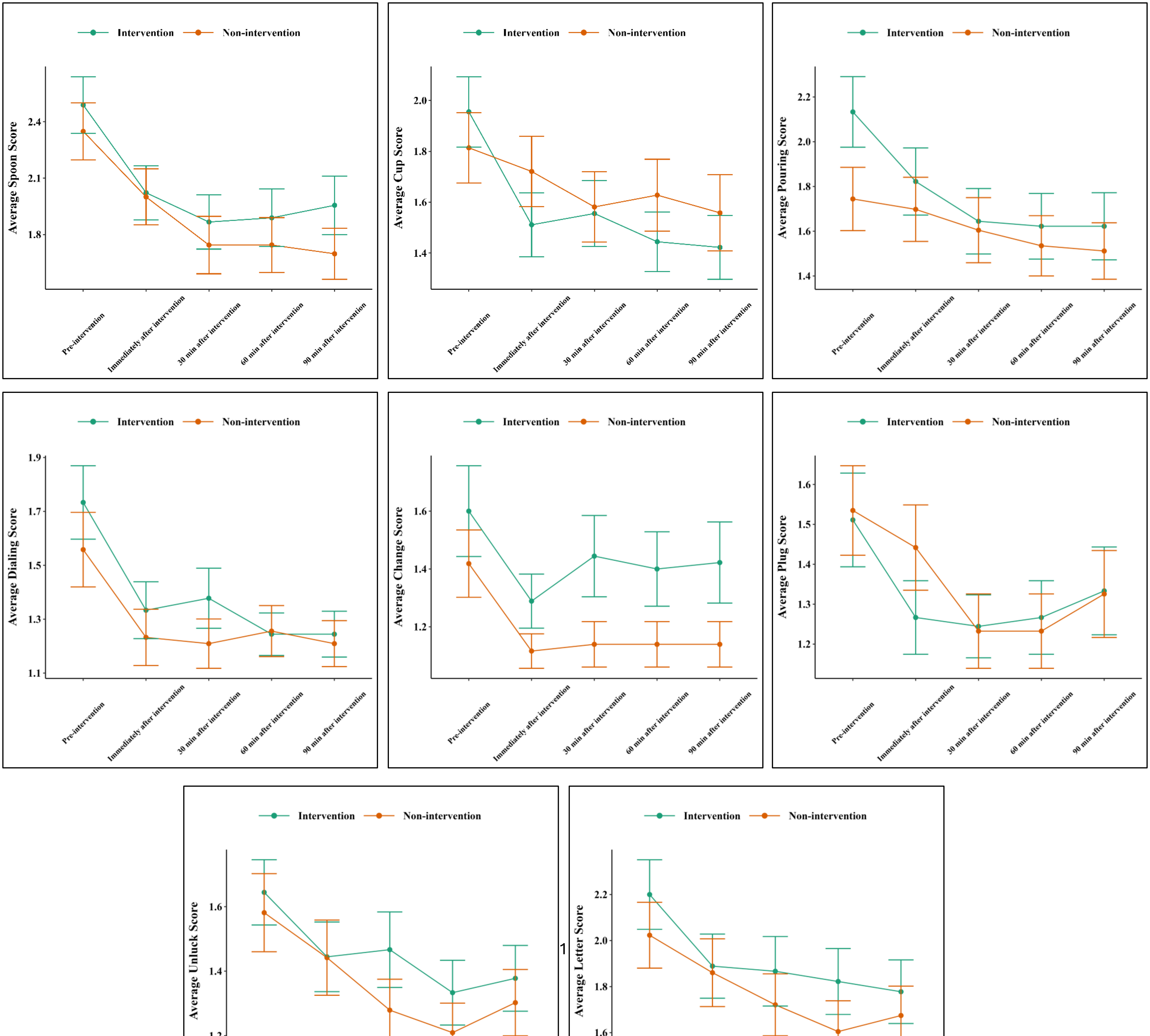

Table S3. Comparison of efficacy outcomes at different follow-up time points among groups

Variables	Group	Baseline	Post-intervention time points									P †	P ‡
		Pre-int	Immediately	30 min	60 min	90 min	3h	4h	5h	6h	24h		
**Tremor amplitude**†	Int	1420.94 ± 239.95	767.74 ± 616.77	859.34 ± 675.90	952.76 ± 651.36	948.80 ± 572.91	----	----	----	----	----	<0.001	----
	Non-int	1334.40 ± 327.05	1227.64 ± 475.78	1159.36 ± 486.84	1228.87 ± 451.85	1207.13 ± 443.58	----	----	----	----	----	0.034	----
**Total TETRAS**	Int	11.11 ± 5.53	8.11 ± 5.32	8.60 ± 5.79	8.64 ± 5.87	8.42 ± 5.91	----	----	----	----	----	<0.001	------
	Non-int	9.86 ± 5.20	8.02 ± 5.42	8.23 ± 5.55	8.07 ± 5.44	7.88 ± 5.60	----	----	----	----	----	<0.001	------
Forward postural	Int	1.80 ± 0.82	1.20 ± 0.69	1.31 ± 0.70	1.24 ± 0.88	1.29 ± 0.82	----	----	----	----	----	<0.001	----
	Non-int	1.74 ± 0.76	1.16 ± 0.92	1.35 ± 0.78	1.35 ± 0.78	1.23 ± 0.81	----	----	----	----	----	0.002	----
Lateral postural	Int	1.76 ± 0.88	1.27 ± 0.89	1.27 ± 0.92	1.42 ± 0.94	1.36 ± 0.88	----	----	----	----	----	0.019	----
	Non-int	1.67 ± 0.84	1.40 ± 0.82	1.42 ± 0.88	1.35 ± 0.92	1.33 ± 0.97	----	----	----	----	----	<0.001	----
Kinetic	Int	1.69 ± 1.10	1.20 ± 1.01	1.33 ± 1.13	1.33 ± 1.09	1.20 ± 1.16	----	----	----	----	----	<0.001	----
	Non-int	1.16 ± 1.15	0.98 ± 0.94	0.91 ± 1.04	0.88 ± 1.01	0.79 ± 1.04	----	----	----	----	----	0.002	----
Spiral drawing	Int	2.11 ± 1.17	1.49 ± 1.20	1.69 ± 1.26	1.60 ± 1.27	1.56 ± 1.25	----	----	----	----	----	<0.001	----
	Non-int	1.93 ± 1.14	1.53 ± 1.24	1.58 ± 1.28	1.56 ± 1.22	1.51 ± 1.24	----	----	----	----	----	<0.001	----
Handwriting	Int	1.96 ± 1.22	1.69 ± 1.35	1.56 ± 1.32	1.62 ± 1.34	1.58 ± 1.31	----	----	----	----	----	0.005	----
	Non-int	1.86 ± 1.25	1.58 ± 1.35	1.56 ± 1.37	1.58 ± 1.22	1.60 ± 1.35	----	----	----	----	----	0.062	----
Dot approximation	Int	1.80 ± 1.38	1.27 ± 1.27	1.44 ± 1.36	1.42 ± 1.25	1.44 ± 1.32	----	----	----	----	----	0.150	----
	Non-int	1.49 ± 1.40	1.37 ± 1.45	1.42 ± 1.42	1.35 ± 1.34	1.42 ± 1.26	----	----	----	----	----	0.488	----
**Total BF-ADL**	Int	15.27 ± 5.89	12.58 ± 5.26	11.66 ± 5.23	12.02 ± 5.60	12.16 ± 5.86	12.19 ± 4.40	11.87 ± 4.64	11.81 ± 4.90	12.13 ± 4.62	12.52 ± 5.00	<0.001	0.046
	Non-int	14.02 ± 4.95	12.51 ± 4.27	12.28 ± 5.38	11.35 ± 4.52	11.42 ± 4.53	11.07 ± 3.90	10.90 ± 4.53	10.31 ± 3.63	10.55 ± 3.36	10.18 ± 3.52	<0.001	<0.001
Spoon using	Int	2.49 ± 1.01	2.02 ± 0.97	1.87 ± 0.97	1.89 ± 1.03	1.96 ± 1.04	----	----	----	----	----	<0.001	----
	Non-int	2.35 ± 1.00	2.00 ± 0.98	1.74 ± 1.00	1.74 ± 0.95	1.70 ± 0.89	----	----	----	----	----	<0.001	----
Cup holding	Int	1.96 ± 0.93	1.51 ± 0.84	1.56 ± 0.87	1.44 ± 0.79	1.42 ± 0.84	----	----	----	----	----	<0.001	----
	Non-int	1.81 ± 0.91	1.72 ± 0.91	1.58 ± 0.91	1.63 ± 0.93	1.56 ± 0.98	----	----	----	----	----	0.025	----
Milk pouring	Int	2.13 ± 1.06	1.82 ± 1.01	1.64 ± 0.98	1.62 ± 0.98	1.62 ± 1.01	----	----	----	----	----	<0.001	----
	Non-int	1.74 ± 0.93	1.70 ± 0.94	1.60 ± 0.96	1.53 ± 0.88	1.51 ± 0.83	----	----	----	----	----	0.030	----
Phone dialing	Int	1.73 ± 0.92	1.33 ± 0.71	1.38 ± 0.75	1.24 ± 0.53	1.24 ± 0.57	----	----	----	----	----	<0.001	----
	Non-int	1.56 ± 0.91	1.23 ± 0.68	1.21 ± 0.60	1.26 ± 0.62	1.21 ± 0.56	----	----	----	----	----	0.027	----
Coin picking up	Int	1.60 ± 1.05	1.29 ± 0.63	1.44 ± 0.94	1.40 ± 0.86	1.42 ± 0.94	----	----	----	----	----	0.247	----
	Non-int	1.42 ± 0.76	1.12 ± 0.39	1.14 ± 0.52	1.14 ± 0.52	1.14 ± 0.52	----	----	----	----	----	0.005	----
Plugging	Int	1.51 ± 0.79	1.27 ± 0.62	1.24 ± 0.53	1.27 ± 0.62	1.33 ± 0.74	----	----	----	----	----	0.268	----
	Non-int	1.53 ± 0.74	1.44 ± 0.70	1.23 ± 0.61	1.23 ± 0.61	1.33 ± 0.72	----	----	----	----	----	0.011	----
Door unlocking	Int	1.64 ± 0.68	1.44 ± 0.73	1.47 ± 0.79	1.33 ± 0.67	1.38 ± 0.68	----	----	----	----	----	0.008	----
	Non-int	1.58 ± 0.79	1.44 ± 0.77	1.28 ± 0.63	1.21 ± 0.60	1.30 ± 0.67	----	----	----	----	----	0.002	----
Letter writing	Int	2.20 ± 1.01	1.89 ± 0.94	1.87 ± 1.01	1.82 ± 0.96	1.78 ± 0.93	----	----	----	----	----	<0.001	----
	Non-int	2.02 ± 0.94	1.86 ± 0.97	1.72 ± 0.88	1.60 ± 0.88	1.67 ± 0.84	----	----	----	----	----	<0.001	----
**CGI-I**	Int	----	2.53 ± 0.89	2.53 ± 0.99	2.49 ± 1.10	2.51 ± 1.14	2.76 ± 1.43	2.86 ± 1.43	2.83 ± 1.44	2.83 ± 1.42	3.20 ± 1.35	0.851	0.003
	Non-int	----	3.02 ± 0.91	2.93 ± 1.12	2.91 ± 1.11	2.72 ± 1.14	3.04 ± 1.21	3.08 ± 1.09	3.08 ± 1.19	3.20 ± 1.12	3.08 ± 1.19	0.020	0.772

Note: Generalized Estimating Equations (GEE) were used to assess the temporal changes in outcomes across the follow-up time points. Symbols † and ‡ represent the p-values derived from GEE analyses, indicating the significance of changes in outcomes from pre-intervention to 90 minutes and 24 hours post-intervention, respectively. *Abbreviations*: BF-ADL: Bain and Findley Activities of Daily Living; CGI-I: Clinical Global Impression-Improvement; Int: intervention group; Non-int: non-intervention group; TETRAS: Essential Tremor Rating Assessment Scale; min: minutes; h: hour. † The sensors of the accelerometer were 16-bit devices, and the sensitivity was between -2G and +2G. G = 9.8 m/s^2

Table S4. Relevant studies investigating median and radial nerve stimulation for tremor

Author, year	Design	Sample size[1]	Efficacy outcomes	Stimulation duration	Time points	Statistical method	Findings
**Lin et al. 2018 [1]**	Sham-controlled pilot trial	23<br>PNS: 10<br>Sham: 13	• TETRAS:<br>- Archimedes spiral drawing<br><br>• Amplitude reduction	a single 40-minute session	• baseline<br>• immediately post-stimulation	Not mentioned	• Spiral drawing:<br>- The response in the treatment group was significant compared with both baseline and sham.<br>- pre- vs. post-stimulation in treatment: 2.77 ± 0.22 vs. 1.77 ±0.21 (**p = 0.01**)<br>- pre- vs. post-stimulation in sham: 2.62 ± 0.14 vs. 2.37 ± 0.22 (p = 0.37)<br><br>• amplitude reduction (estimated based on the response to treatment) in treatment vs. sham: 60% ± 8.4% vs. NA (**p = 0.02**)
**Pahwa et al. 2019 [2]**	RCT	77<br>PNS: 40<br>Sham: 37	• TETRAS tasks:<br>- Archimedes spiral<br>- Forward postural<br>- Lateral postural<br>- Kinetic<br><br>• BF-ADL tasks:<br>- Spoon using<br>- Cup holding<br>- Milk pouring<br>- Phone dialing<br>- Coin picking up<br>- Plugging<br>- Door unlocking<br><br>• CGI-I	a single 40-minute session	• baseline<br>• immediately post-stimulation	• ANCOVA[2]: Archimedes spiral score<br><br>• Independent-Samples T-test [3]: other TETRAS tasks and BF-ADL<br><br>• Wilcoxon Rank Sum test [4]: CGI-I	• TETRAS (improvement in treatment vs. sham):<br>- Archimedes spiral: p = 0.26<br>- Forward postural: **p = 0.004**<br>- Lateral postural: NS<br>- kinetic: NS<br>- Combined upper limb[5]: **p = 0.017**<br><br>• BF-ADL (improvement in treatment vs. sham):<br>- Cup holding: **p = 0.011**<br>- Phone dialing: **p = 0.015**<br>- Coin picking up: **p = 0.002**<br>- Door unlocking: **p = 0.010**<br>- Spoon using: NS<br>- Milk pouring: NS<br>- Plugging: NS<br><br>- The treatment group improved significantly compared to the baseline on all seven activities. The sham group improved significantly compared to the baseline for five of the seven activities [6].<br><br>- Treatment improved ADLs across all measured tasks by 0.66 while sham improved by 0.36 (**p = 0.001**)<br><br>• CGI-I:<br>- Treatment subjects reported improvement in the CGI-I scale that was significantly greater than sham (**p = 0.019**).<br>- a greater percentage of subjects in the treatment group reported an improvement after stimulation (88%), which was a significant improvement compared to the sham group (62%) (**p = 0.019**).
**Isaacson et al. 2020 [3]**	open-label, post-clearance, single-arm clinical trial	205	• TETRAS<br>- Forward postural<br>- Lateral postural<br>- Kinetic<br>- Archimedes spiral<br>- Handwriting<br>- Dot approximation<br><br>• BF-ADL<br>- Spoon using	twice daily (40 minutes each) for three months	• baseline<br>• one-month post-stimulation<br>• three-month post-stimulation	• Independent-Samples T-test: TETRAS, BF-ADL, QUEST<br><br>• Wilcoxon Rank Sum test: power	• TETRAS and BF-ADL scores improved from baseline to three-month post-stimulation.<br>• Patients showed improvement in TETRAS and BF-ADL from pre- to post-stimulation at each in-clinic visit (**p < 0.0001**).<br>• Tremor power improved during home use, with the mean tremor power over all patients decreasing from 1.1 ± 0.3 $(m/s^2)^2$ pre-stimulation to 0.3 ± 0.1 $(m/s^2)^2$ post-stimulation (**p < 0.0001**).

[1] For efficacy analysis
[2] ANCOVA was used to assess statistical significance of the difference in the mean change between the groups for TETRAS Archimedes spiral score. The model included the baseline score for the task as a continuous covariate, and randomization assignment as a classification variable.
[3] Independent-Sample T test was used to assess significance of the differences in the mean change between the groups in other TETRAS tasks and BF-ADL.
[4] Wilcoxon Rank Sum test was used to assess significance of the differences in the mean change between the groups in CGI-I.
[5] Combination of forward postural, lateral postural, and kinetic assessments.
[6] using a spoon to drink soup, holding a cup of tea, pouring milk from a bottle or carton, dialing a telephone, and inserting an electric plug into a socket.

			- Cup holding<br>- Milk pouring<br>- Phone dialing<br>- Coin picking up<br>- Plugging<br>- Door unlocking<br><br>• CGI-I<br>• PGI-I<br>• QUEST survey<br>• Tremor power				• In QUEST surveys conducted after three months of use, patients indicated their quality of life improved (–3.1 ± 0.9 change (**p = 0.0019**).
**Yu et al. 2020 [4]**	single-arm, open-label study	15	• FTM-CRS<br>- Postural hold<br>- Spiral drawing<br>- Finger-to-nose reach<br>- Pouring<br><br>• Reduction in accelerometer-measured tremor power relative to baseline for each task.	a single 40-minute session	• baseline<br>• immediately after 30- and 60-min post-stimulation	• binomial test	• The mean FTM-CRS scores improved for at least 60 min beyond the end of TAPS for 80% (12 of 15 patients) (**p = 4.6e–9**)<br>• Similarly, for each assessed task, tremor power improved for at least 60 min beyond the end of TAPS for over 70% of patients.<br>• The postural hold task had the largest reduction in tremor power (median 5.9-fold peak reduction in tremor power) and had at least 60 min of improvement relative to baseline beyond the end of TAPS therapy for 73% (11 of 15, **p = 9.8e–8**) of patients.
**Brillman et al. 2022 [5]**	retrospective post-market surveillance	216 (9,163 sessions)	• improvements in tremor power[7] from before to after a therapy session	5.4 ± 4.5 times per week	• baseline<br>• after at least 10 sessions (within 90 to 663 days)	• Wilcoxon sign-rank test	• TAPS reduced tremor power by 71% (geometric mean) across all sessions, with 59% of patients experiencing >50% tremor reduction after their sessions.<br>• Eighty-four percent (84%) of patients who returned the voluntary survey reported improvement in at least one of eating, drinking, or writing, and 65% of patients reported improvement in quality of life.<br>• Analysis by quartiles of tremor severity showed patients experienced the greatest tremor reductions when they used TAPS when their tremors were most severe.
**Lu et al. 2023 [6]**	retrospective post-market analysis	808 (36,411 sessions)	• tremor power improvement ratio<br>• percentage of tremor reduction<br>• percentage of sessions rated as improved	average 5.6 sessions per week	• baseline<br>• after chronic use [8]	• linear mixed effects regression: pre-stimulation tremor severity effect on improvement<br><br>• linear mixed effects regression: potential habituation of TAPS effects [9]	• The average (geometric mean) tremor power improvement ratio across all patients was 2.8 (arithmetic mean, 710.5; median, 2.1), i.e., a 64.3% reduction in tremor power.<br>• 49.8% of patients showed at least 50% of tremor reduction, and 88.1% of patients had at least 50% of sessions with a tremor improvement ratio greater than 1.<br>• A significant main effect of pre-stimulation tremor severity was observed.<br>• Specifically, patients with the high quartile tremor severity in both within-subject (average improvement ratio: 9.6; 95% CI, 8.8 to 10.4) and between-subject (average improvement ratio: 7.5; 95% CI, 5.8 to 7.8) classification experienced the most tremor improvement compared with patients with the low or medium tremor severity (p < 0.001 for all post-hoc pairwise comparisons in both classifications).<br>• On average, 60.5% of sessions were rated as improved. The percent of sessions rated as improved were comparable across the within-subject classification (about 60%) while the average percentages were greater in high and medium groups compared with the low group in the between-subject classification.<br>• Approximately 62% of survey respondents either had reduced medication or planned to consult physicians about their medication usage.

[7] computed from device postural hold accelerometry data
[8] Patients had used therapy between 90 and 1,233 days.
[9] how tremor improvement varied over time

							• No habituation was observed in TAPS effectiveness in long-term patients

Abbreviations: BF-ADL: Bain and Findley Activities of Daily Living; CGI: Clinical Global Impression-Improvement; FTM-CRS: Fahn-Tolosa-Marin Clinical Rating Scale; Patient Global Impression scores; QUEST: Quality of Life in Essential Tremor; TAPS: transcutaneous afferent patterned stimulation; TETRAS: Essential Tremor Rating Assessment Scale.

References:

1. Lin, P.T., E.K. Ross, P. Chidester, et al., *Noninvasive neuromodulation in essential tremor demonstrates relief in a sham-controlled pilot trial.* Mov Disord, 2018. **33**(7): p. 1182-1183.
2. Pahwa, R., R. Dhall, J. Ostrem, et al., *An Acute Randomized Controlled Trial of Noninvasive Peripheral Nerve Stimulation in Essential Tremor.* Neuromodulation, 2019. **22**(5): p. 537-545.
3. Isaacson, S.H., E. Peckham, W. Tse, et al., *Prospective Home-use Study on Non-invasive Neuromodulation Therapy for Essential Tremor.* Tremor Other Hyperkinet Mov (N Y), 2020. **10**: p. 29.
4. Yu, J.Y., A. Rajagopal, J. Syrkin-Nikolau, et al., *Transcutaneous Afferent Patterned Stimulation Therapy Reduces Hand Tremor for One Hour in Essential Tremor Patients.* Front Neurosci, 2020. **14**: p. 530300.
5. Brillman, S., K. Colletta, S. Borucki, et al., *Real-World Evidence of Transcutaneous Afferent Patterned Stimulation for Essential Tremor.* Tremor Other Hyperkinet Mov (N Y), 2022. **12**: p. 27.
6. Lu, C., D. Khosla, A. Kent, et al., *Transcutaneous Afferent Patterned Stimulation for Essential Tremor: Real-World Evidence with Long Term Follow-Up.* Tremor Other Hyperkinet Mov (N Y), 2023. **13**: p. 29.
